# Transcription factor clusters enable target search but do not contribute to target gene activation

**DOI:** 10.1093/nar/gkad227

**Published:** 2023-03-29

**Authors:** Joseph V W Meeussen, Wim Pomp, Ineke Brouwer, Wim J de Jonge, Heta P Patel, Tineke L Lenstra

**Affiliations:** Division of Gene Regulation, The Netherlands Cancer Institute, Oncode Institute, 1066CX Amsterdam, The Netherlands; Division of Gene Regulation, The Netherlands Cancer Institute, Oncode Institute, 1066CX Amsterdam, The Netherlands; Division of Gene Regulation, The Netherlands Cancer Institute, Oncode Institute, 1066CX Amsterdam, The Netherlands; Division of Gene Regulation, The Netherlands Cancer Institute, Oncode Institute, 1066CX Amsterdam, The Netherlands; Division of Gene Regulation, The Netherlands Cancer Institute, Oncode Institute, 1066CX Amsterdam, The Netherlands; Division of Gene Regulation, The Netherlands Cancer Institute, Oncode Institute, 1066CX Amsterdam, The Netherlands

## Abstract

Many transcription factors (TFs) localize in nuclear clusters of locally increased concentrations, but how TF clustering is regulated and how it influences gene expression is not well understood. Here, we use quantitative microscopy in living cells to study the regulation and function of clustering of the budding yeast TF Gal4 in its endogenous context. Our results show that Gal4 forms clusters that overlap with the *GAL* loci. Cluster number, density and size are regulated in different growth conditions by the Gal4-inhibitor Gal80 and Gal4 concentration. Gal4 truncation mutants reveal that Gal4 clustering is facilitated by, but does not completely depend on DNA binding and intrinsically disordered regions. Moreover, we discover that clustering acts as a double-edged sword: self-interactions aid TF recruitment to target genes, but recruited Gal4 molecules that are not DNA-bound do not contribute to, and may even inhibit, transcription activation. We propose that cells need to balance the different effects of TF clustering on target search and transcription activation to facilitate proper gene expression.

## INTRODUCTION

Gene-specific transcription factors (TFs) are essential for correct control of gene expression. Eukaryotic TFs contain a DNA-binding domain (DBD) which binds to specific sequences in regulatory promoter and enhancer regions, and a transactivation domain (AD) that interacts with cofactors, chromatin remodelers and other transcriptional regulators to facilitate transcription (1). Already >25 years ago, it was observed that the glucocorticoid receptor TF was not homogeneously distributed through the nucleus, but forms areas of high local concentration (2), referred to as clusters, hubs, condensates or droplets. These clusters have since been observed for many other TFs, cofactors and RNA polymerase II (3–11). Despite the widespread observation of clustering, the regulation and function of TF clustering is not well understood.

It has been suggested that cluster formation is driven by multivalent interactions between intrinsically disordered regions (IDRs) (4,12,13). IDRs are enriched in the transactivation domains of many TFs and enable self-interactions (homotypic interactions) and multivalent interactions with IDRs of other components of the transcriptional machinery (heterotypic interactions) (4,11,12,14–16). Support for the role of IDRs in TF cluster formation comes from the finding that interactions between the IDRs of TFs and Mediator are important for TF cluster formation *in vitro* (4,17). However, clustering *in vivo* can also occur independently of IDRs, such as for Sox2 and the glucocorticoid receptor (18,19). For Sox2, clustering is mostly dependent on DNA binding (18), suggesting that these clusters reflect binding to adjacent motifs in the genome rather than protein-protein interactions. How endogenous TF clusters are regulated by IDRs, the configuration of binding sites and interactions with DNA or other regulators is only starting to emerge (11,17,20).

Moreover, an important open question is how clustering influences TFs during the different steps of transcription activation (13,21–23). Clustering and IDR-mediated interactions have been reported to enhance target search (increasing the DNA binding rate) (24–28), to increase the local concentration of TFs at the promoter (increasing the DNA binding rate), to stabilize TF binding to DNA (decreasing the rate of DNA unbinding) (12), to enable 3D genomic interactions between target genes (29) and to boost transcription activation through enhanced recruitment of cofactors and polymerase molecules (30,20,16,31,32). In contrast, in some cases TF clustering can inhibit gene expression, as shown for synthetic TFs and the oncogenic TF EWS::FLI1 (33,34). These discrepancies illustrate our lack of understanding of how clustering impacts transcription. Although novel inducible artificial clustering tools provide precise control of clustering, it remains unclear how these results can be extrapolated to endogenous gene regulation.

Here, we used the transcription factor Gal4 from budding yeast to study the regulation and function of TF clustering in an endogenous context. The expression and activity of this TF are regulated by different carbon sources (35). In the presence of glucose, *GAL4* and its target genes are transcriptionally repressed by Mig1 in a concentration dependent manner (36). In addition, if Gal4 is expressed but galactose is absent, for example when raffinose is the sole carbon source, the activity of the Gal4 protein is inhibited by binding to its inhibitor Gal80 (37). In the presence of galactose, Gal80-mediated inhibition is relieved and Gal4 activates the expression of the *GAL* genes to metabolize galactose. The naturally low protein levels of Gal4 and the small number of Gal4 target genes make Gal4 an excellent model to study the effects of TF clustering on transcription at a single locus in an endogenous context.

Using quantitative live-cell imaging of Gal4-EGFP, we find that Gal4 forms clusters *in vivo* that colocalize with target genes. Gal4 cluster abundance, size and density change across different growth conditions, are dependent on the Gal4 expression levels and are limited by interactions with the inhibitor Gal80. Removal of endogenous Gal4 binding sites and analysis of truncation mutants showed that both DNA binding and IDRs contribute to, but are not essential for Gal4 clustering. In addition, regions outside of the Gal4 DNA binding domain are sufficient to recruit additional Gal4 molecules to clusters at target genes, indicating that self-interactions between Gal4 molecules facilitate target search. However, non-DNA-bound Gal4 molecules present in a cluster at a target locus do not necessarily contribute to transcription and might even inhibit transcription. Taken together, we propose that clustering positively affects target search and negatively affects transcription activation, and these aspects therefore need to be properly balanced to facilitate gene expression.

## MATERIALS AND METHODS

### Yeast strains and plasmids

All strains were derived from BY4741 and BY4742 parent strains. The BY4742 *GAL4-EGFP* strain (YTL390) was created by transformation using a PCR product with EGFP and *loxP-kanMX-loxP* followed by kanMX removal by CRE recombinase. The BY4742 *gal4Δ* strain (YTL559) was created by transformation using a PCR produced containing a kanMX cassette.

Truncations and mutations of *GAL4* were created using a CRISPR-Cas9-based approach (38): *BPSV40-GAL4(Δ1–94)* (Gal4ΔDBD; YTL1284 and YTL1662), *GAL4(Δ840–881)* (Gal4ΔminiAD; YTL1221), *GAL4(Δ768–881)* (Gal4ΔAD; YTL1286), *GAL4(Δ95–881)* (Gal4-DBD-only; YTL1226, YTL1639 and YTL1686), *GAL4::BPSV40* (YTL1702) and *GAL4(S41D)* (YTL945). Strains were transformed using a plasmid expressing Cas9 and a guide RNA and either double-stranded PCR repair template or single-stranded oligo, followed by removal of the Cas9 plasmid by 5-FOA selection.

To scramble the Gal4 UAS sites (scrUAS) at *pGAL2*, *pGAL7* and *pGAL1-10* (YTL1154), three successive rounds of transformations were used to edit one locus at a time using the CRISPR-based approach described above with single-stranded oligos as repair templates.

To introduce the DNA label at the *GAL* locus (*3′-GAL1*; YTL1652, YTL1662 and YTL1686), *3′-RNR2* (YTL1699) or *5′-RNR2* (YTL1698), three successive rounds of transformations were used, as described in (39). First, a natMX cassette was integrated at either *3′-GAL1*, *3′-RNR2* or *5′-RNR2* by transformation with a PCR product encoding for natMX and homology arms for the *tetO* array. Next, the natMX cassette was replaced with *tetOx128* array using the CRISPR-based approach described above using a PCR product as repair template. Finally, *tetR1-tdTomato* was integrated at the *ADE1* locus by transformation using a plasmid digestion as a repair template. The BY4743 strains with the DNA label at the *GAL* locus (YTL1678 and YTL1693) were created by mating the BY4742 strain with *GAL* DNA label and *GAL4-EGFP* or *BPSV40-GAL4(Δ1–94)-EGFP* with either a BY4741 *Δgal4* strain (YTL1679), a WT BY4741 strain (YTL1678) or a BY4741 *GAL4(Δ768–881)* strain (YTL1693).

The BY4743 diploid strains with PP7 loops (YTL1218, YTL1317 for cluster-RNA-label overlap, YTL1098, YTL1326 for growth assay and YTL590, YTL1431 and YTL1432 for live-cell imaging) were created by mating of a BY4741 and a BY4742 haploid yeast strain. The BY4742 strain was either *GAL4-EGFP*, *BPSV40-GAL4(Δ1–94)-EGFP* or *gal4Δ*. The BY4741 strain contained *14xPP7-GAL10*, inserted by transformation with a PCR product containing the PP7 loop cassette and *loxP-kanMX-loxP* followed by *kanMX* removal by CRE recombinase. The PP7 coat protein was inserted in the BY4741 strain by transformation with a digested plasmid as a repair template (pTL174:PacI for PCP-GFPEnvy in YTL590, YTL1431 and YTL1432 or pTL306:PacI for PCP-ymScarletI in YTL1317, YTL1326 and YTL1218).

The BY4742 with *GAL4-EGFP* + *gal80Δ* strain (YTL762) was created by transformation of the BY4742 with *GAL4-EGFP* strain with a PCR product containing a *loxP-kanMX-loxP* cassette to replace *GAL80* and subsequent *kanMX* removal by CRE recombinase. For the BY4742 with *GAL4-EGFP* and *med15Δ* (YTL1304), the *med15Δ* was created using the CRISPR-based approach described above using a single-stranded oligo as a repair template. A plate-based growth assay indicated that YTL762 has a functional galactose metabolism, whereas YTL1304 has not, as expected (Supplementary Figure S3A).

For Western Blot experiments, V5-tags (3x V5) were introduced using the CRISPR-based approach described above using a PCR product as a repair template, using YTL390, YTL762, YTL1284, YTL1221, YTL1286 and YTL1226 as parent strains for YTL1653, YTL1685, YTL1655, YTL1661 and YTL1654, respectively.

The BY4743 diploid strains for RT-qPCR experiments (YTL1834, YTL1835, YTL1836 and YTL1837) were created by mating by mating of a BY4741 and a BY4742 haploid yeast strain containing the appropriate *GAL4* genotype, either *GAL4* (WT), *gal4Δ* (Δ), *GAL4(Δ768–881)* (ΔAD) or *BPSV40-GAL4(Δ1–94)* (ΔDBD).

For all strains at least two replicates were constructed independently, which were verified by PCR and, if applicable, sequencing. All strains, plasmids and oligos used in this study are listed in Supplementary Tables S1, S2 and S3, respectively. Yeast strains and plasmids are available on request.

### Live-cell imaging of Gal4 clustering

Yeast cultures were started in synthetic complete medium in the morning, diluted in the evening and grown overnight (O/N) to mid-log (OD_600 nm_ 0.2–0.4) whereafter they were imaged on a coverslip with a 2% agarose pad, as described previously (40). For all experiments, unless indicated otherwise in the legends, the indicated carbon sources were present throughout the entire experiment. For cells with a DNA-label, containing the *ade1::tetR1-tdTomato-kanMX* integration, 40 mg/L adenine was added to both synthetic complete medium and agarose pad to rescue *ade1* deficiency.

Imaging was performed on an AxioObserver.7 / ELYRA.P1 microscope (Zeiss) equipped with an incubator for microscopy (Pecon) set at 30°C, Scanning Stage Piezo 130 × 100 (Zeiss) and 405, 488, 561 and 640 nm lasers (Coherent) with maximum powers at 50, 100, 100 and 150 mW respectively. We used an alpha Plan-Apochromat 100× NA 1.57 oil objective (Zeiss), and a filterset consisting of a ZT405/488/561/640rpcv2-UF1 dichroic filter (Chroma) and a ZET405/488/561/640mv2 emission filter (Chroma). The emission was split in two channels (TV1 and TV2) using a duolink splitter (Zeiss) holding a filterset with a BS561 dichroic beamsplitter (Zeiss) and FF03-525/50–25 and BLP02-561R-25 emission filters (Semrock) used for imaging DNA- or RNA-labels and Gal4-EGPF clusters respectively on two EM-CCD iXon DU 897 camera's (Andor). All imaging was performed using the following settings in Zen Blue software: TIRF acquisition mode, 512 × 512 pixels field of view, 1.6× optovar, HILO illumination mode, 50 ms exposure time, EMCCD gain set to 100× and *z*-stacks (using the piezo) were set to 21 planes at 250 nm intervals.

The Gal4-EGFP clusters were imaged with excitation at 488 nm at 25% power resulting in resulting in a ±2 kW/cm^2^ excitation intensity. When imaging either the DNA-label or RNA-label, an extra *z*-stack (TV1) was taken prior to the *z*-stack capturing the clusters (TV2), using excitation at 561 nm at 0.2% power for the DNA label (±16 W/cm^2^ excitation intensity) and 0.1% power for the RNA-label (±8 W/cm^2^ excitation intensity).

Replicates of conditions to be compared were always imaged on the same day and comparisons were only made between these (paired-)replicates.

### Image segmentation

Clustering microscopy data was analyzed using custom Python software (10.5281/zenodo.7650154 with dependencies from 10.5281/zenodo.7650168 and 10.5281/zenodo.7650172). For all experiments, the EGFP channel (TV2) was used for cell detection and image segmentation. Briefly, a maximum intensity projection of the 3D *z*-stack was made which was then smoothed using a gaussian filter with *σ* 2.5 voxels. The resulting image was then thresholded using Otsu's method to find areas containing cells. Any holes in this mask were filled and small features were removed. Finally, the cells were separated using watershedding with local maxima at least 40 pixels apart functioning as starting points. Then the area of the nucleus was determined for each cell individually by thresholding the cell. The threshold was determined by Otsu's method on the 75% of brightest non-zero pixels. The part of the cell higher than this threshold was taken to be the nucleus. After this automated segmentation, the cellular and nuclear masks were checked manually and corrected when multiple cells were masked together or when a mask contained debris or a dead cell. Additionally, for RNA-label-overlap experiments, cells without any transcription site were removed as their bright nuclei with high levels of fluorescently labelled coat protein led to background false-positive transcription sites being picked up during spot detection.

### Spot detection

Initial spot detection was done by applying a difference of gaussians filter (DoG) to the 3D *z*-stack. The DoG filter was applied with *σ* 2.075 and 1.245 pixels in *x* and *y* directions and 1.325 and 0.795 planes in *z* direction. The local maxima found were considered to be spot candidates.

Various absolute thresholds were used to select only local maxima above the background. These thresholds were kept the same for all conditions and replicates which were compared directly.

### Spot fitting

A region of interest (ROI) of 11 pixels in *x* and *y* directions and seven planes in *z* direction was cut out of the image around each spot candidate. Then the parameters of this spot were determined in two steps: (i) iterative moment analysis and (ii) fitting.

We assume each spot can be described by a gaussian profile on top of a tilted background:


$$\begin{eqnarray*} && G\ \left( {x,y,z} \right) = \frac{I}{{{{\left( {2\pi } \right)}}^{3/2}\sigma _{xy}^2{\sigma }_z}}\nonumber\\ && \quad {\rm{\ \cdot \exp}}\left[ { - \frac{{{{\left( {x - {x}_0} \right)}}^2 + {{\left( {y - {y}_0} \right)}}^2}}{{2\sigma _{xy}^2}} - \frac{{{{\left( {z - {z}_0} \right)}}^2}}{{2\sigma _z^2}}} \right]\nonumber\\ && \quad +\, b + {b}_xx + {b}_yy + {b}_zz\end{eqnarray*}$$

To ensure that we are calculating the parameters of the spot candidate instead of a nearby neighbor, the image in the ROI is multiplied by a gaussian approximating the microscope point spread function (psf, ${\sigma }_{xy}$ = 1.66 pixels and ${\sigma }_z$ = 1.06 pixels), centered in each iteration on the location ${x}_0,{y}_0,{z}_0$ of the spot determined by moment analysis in the step before. Besides the location, the background $b,{b}_x,{b}_y,{b}_z$ is determined in each iteration by fitting linear functions through the voxels at the edges of the ROI. This process is stopped when either the location does not significantly improve anymore between iterations, when the new location is more than 3 voxels from the spot candidate location or when 20 or more iterations were performed. Thereafter, the spot intensity $I$ and width ${\sigma }_{xy}, {\sigma }_z$ are determined by moment analysis, correcting for the limited domain of the ROI as moment analysis expects an infinite domain. The resulting parameters were then used as an initial guess for a Limited-memory Broyden–Fletcher–Goldfarb–Shanno (L-BFGS-B) optimization on the sum of weighted squared log residuals, again to make sure that the spot candidate is fitted. The weight is defined as a gaussian with ${\sigma }_{xy}$ = 3.32 pixels and ${\sigma }_z$ = 2.12 pixels centered on the last position determined by the moment analysis. Subsequently, the goodness of fit is determined as the adjusted coefficient of determination for both the fit as a whole and for the peak and background parts individually. Finally, the peak intensity ${I}_p$, defined as the height of the maximum of the gaussian fit above the background, was calculated as:


$$\begin{equation*}{I}_p = \frac{I}{{{{( {2\pi } )}}^{3/2}\sigma _{xy}^2{\sigma }_z}}\end{equation*}$$

### Spot filtering

Spots of which the resultant location was not within 3 voxels from the initial guess were considered to have a failed localization and removed from the results. Additionally, of peaks closer to each other than 0.1 times the psf size (*σ_xy_* = 1.66 pixels and *σ_z_* = 1.06 pixels), only the first was kept. Finally, only spots residing within the cellular masks and of which the goodness of fit (adjusted *R^2^*) of the peak was above –1 were taken into account in the analysis of clusters and DNA-/RNA-labels.

### Quantification of clusters

The frequency of number of clusters per cell was quantified by counting the number of filtered spots within every cell mask, combining the counts of every replicate per condition and thereafter making a normalized histogram of these counts in which the error bars represent the bootstrapped standard error of the mean (1000 repeats).

To compare cluster *σ*, density (*I*_p_) and total intensity (*I*) between conditions, the filtered spots of all replicates within a condition were combined and were shown using boxplots in which the box indicates the quartiles of the dataset and the whiskers extend to 1.5 times the inter-quartile range. The boxplots were overlayed with the individual data points. For visualization purposes, the axis range was chosen such that it was easy to compare the boxplots between conditions. Note that in some cases, this led to a few single datapoints ending up outside of the displayed range of the plots. We note that all values, also those outside the displayed range, were included during statistical testing.

For both the number of clusters per cell, cluster *σ*, density and total intensity, the differences in population median between multiple conditions were first tested using the Kruskal–Wallis *H*-test for independent samples, followed by two-sided pairwise Mann–Whitney *U* tests.

### Quantification of colocalization between clusters and the DNA-/RNA-label

On every day of two-channel imaging, a *z*-stack of 0.21 μm TetraSpeck™ microspheres (ThermoFisher) was made to correct for aberrations between the channels of the DNA-/RNA-label (hereafter named ‘reference label’; TV1) and clusters (TV2). In brief, a 2D affine transformation mapping TV2 to TV1 was determined using SimpleElastix and max *z* projections of both channels of the two-color bead sample, correcting for aberrations in *x* and *y*. For *z*, we assumed a simple (focus) offset, which was determined by processing the bead sample using the same spot detection and fitting pipeline as for the clusters, with the minor modification of using a 10× standard deviation threshold for determining the local maxima. We then found the nearest neighbor pairs between channels. These pairs were filtered from outliers using the interquartile range rule and the offset in the *z* direction was determined as the mean distance between the beads in each pair in *z*. Finally, the locations of the spots in the second channel (TV2) where corrected using the affine transformation and the offset in z prior to distance calculations.

After spot filtering, the reference label of every cell was determined as the spot with the largest density (*I*_p_) within the nuclear mask, as both the DNA- and RNA-labels are expected to be in the nucleus. Within each reference label-containing cell, all 3D and 2D cluster-reference distances were calculated. The distributions of the beforementioned spot parameters (*σ*, density and total intensity) of spots with a distance closer or further than the overlap threshold were tested for differences using the two-sided Mann–Whitney *U* test.

For the calculation of fraction of nearest neighbor distances (NNDs) all cells with a reference label were taken into account. For every cell, the NND was determined as the minimal 3D/2D distance. Cells with a reference label but lacking clusters were also included as fractions were normalized to all cells containing a reference label, but their NNDs were set to ‘nan’. Finally, the NNDs were divided over 0.1 μm bins in a histogram normalized to all cells with a reference label. The standard error of the mean of every bin was calculated using bootstrapping (1000 repeats). Differences between conditions in populations of cells with an overlapping cluster-reference label were calculated using a two-sided Fisher's exact test.

### Quantification of $\sigma$versus density

To test whether the spot fitting algorithm can independently fit spot *σ* and density, *z*-stacks were taken of fluorescent beads (0.21 μm TetraSpeck microspheres, ThermoFisher) at different laser powers. Apart from the varying laser powers, imaging settings were as before. Spots were localized with a standard deviation threshold of 10 for the detection of local maxima. Spots for which localization failed were filtered out as before. As expected, the *σ* stays constant while the spot density changes at different laser powers (Supplementary Figure S1B–D).

### Fit of $\sigma$ versus diameter

To estimate the relationship between the measured $\sigma$ and the spot diameters, *z*-stacks were taken of 100, 200, 500 and 1000 nm fluorescent beads (TetraSpeck™ microspheres, Invitrogen T14792). Imaging settings were as before, with the minor modification that an alpha Plan-Apochromat 100× NA 1.46 oil objective was used as this corresponded to the coverslip on which the beads were mounted. Spots were localized with an adjusted DoG filter for NA 1.46 and a standard deviation threshold of 10 for the detection of local maxima. Spots for which localization failed were filtered out as before. The relationship between the $\sigma$ (Supplementary Figure S1E), and the diameter $d$ was described empirically as:


$$\begin{equation*}d\ = {\rm{\ }}{\left( {\frac{{{\sigma }^\beta - \sigma _0^\beta }}{\alpha }} \right)}^{\frac{1}{\beta }}\end{equation*}$$

**Figure 1.**
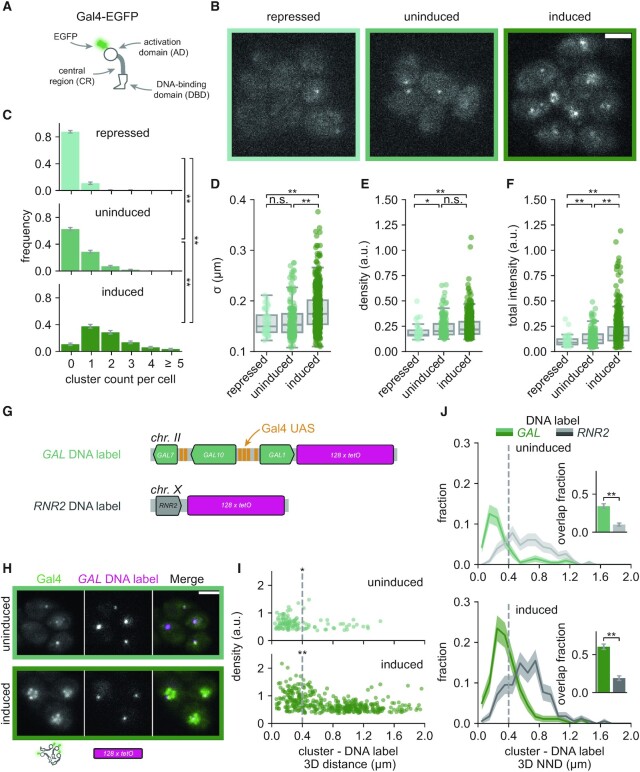
Gal4 forms clusters that colocalize with the *GAL* genes. (**A**) Schematic representation of Gal4. The activation domain (AD), central region (CR) and DNA-binding domain (DBD) are indicated. For visualization, the C-terminus of the endogenous Gal4 is tagged with EGFP (green). Note that Gal4 binds DNA as a dimer. (**B**) Representative images of Gal4-EGFP clusters in yeast cells grown in repressed (glucose, very light green), uninduced (raffinose, light green) and induced (galactose, green) conditions. Images are a single *z*-slice of a representative group of cells. Scalebar: 3 μm. (C–F) Quantification of Gal4-EGFP clusters in repressed (very light green, 285 cells), uninduced (light green, 381 cells) and induced (dark green, 280 cells) conditions. (**C**) Distribution of number of clusters observed per cell. Error bars indicate standard error of the mean (SEM) based on 1000 bootstrap repeats. (D–F) Distribution of (**D**) cluster *σ*, (**E**) density and (**F**) total intensity, represented by the sigma, peak height and integrated intensity of the 3D gaussian fit, respectively (see Materials and Methods for details). Circles show data for individual clusters and box plots show the distribution of the data, with box edges indicating first and third quartiles, center line indicating the median and whiskers indicating the 1.5× interquartile range. Significance determined by Mann–Whitney *U* test; n.s.: not significant; *P < 0.05; **P < 0.01. (**G**) Schematic representation of genomic integration of the *128xtetO* DNA label (magenta) at the *GAL* locus (light green), with six Gal4 bindings sites (Upstream Activating Sequences, UASs; orange), or at the *RNR2* locus (grey), without Gal4 binding sites. (**H**) Representative images of dual-color fluorescence imaging to determine the colocalization between Gal4-EGFP clusters (green) and the *GAL* locus (magenta) in uninduced (raffinose) and induced (galactose and raffinose) conditions. Images are a single *z*-slice of a representative group of cells. Scalebar: 3 μm. (**I**) Scatterplot of Gal4-EGFP cluster density versus 3D distance between the cluster and the *GAL* DNA label for uninduced (top, dark green) and induced (bottom, light green) conditions (306 and 206 cells, respectively). Vertical dashed line indicates 400 nm threshold used to discriminate between overlapping and non-overlapping clusters. Significance between clusters closer and further than 400 nm from the DNA label was determined by Mann–Whitney *U* test; *P < 0.05; **P < 0.01. (**J**) Distribution of 3D nearest neighbor distances (NND) between the *GAL* (green) and *RNR2* (grey) DNA label and the closest Gal4-EGFP cluster in uninduced (top, light green and light grey, 306 and 276 cells, respectively) and induced conditions (bottom, dark grey and dark green, 200 and 198 cells, respectively). Shaded regions represent SEM based on 1000 bootstrap repeats. Vertical dashed line indicates 400 nm threshold used to discriminate between overlapping and non-overlapping clusters. Inset shows fraction of *GAL* or *RNR2* loci with an overlapping cluster. Error bars represent SEM based on 1000 bootstrap repeats. Significance determined by Fisher's exact test; n.s.: not significant; **P < 0.01.

Fitting this equation to the bead diameters and the mean $\sigma$ for each bead diameter resulted in ${\sigma }_0 = {\rm{\ }}$0.118 ± 0.014 μm, $\alpha \ =$ 0.05 ± 0.05 μm and $\beta \ = {\rm{\ }}$3.46 ± 2.15. To convert the NA 1.46 results to NA 1.57, the acquired ${\sigma }_0{\rm{\ }}$ was multiplied with the ratio 1.46/1.57, giving ${\sigma }_0 =$ 0.109 ± 0.012 μm (Supplementary Figure S1F). Using these parameters, we converted our measurements of $\sigma$ for Gal4-EGFP clusters in induced (galactose) conditions (Figure 1D) to estimate the cluster diameter to be in the range of 100–763 nm. For this conversion, we used the 1.5× interquartile range as minimum and maximum values for $\sigma$.

### Live-cell imaging of transcription dynamics

Live-cell imaging of transcription dynamics was performed as previously described in detail with minor modifications (40–42). In brief, yeast cultures were started in synthetic complete medium containing 2% raffinose in the morning, diluted in the evening and grown O/N to mid-log (OD_600 nm_ 0.2–0.4) whereafter they were imaged on a coverslip with a 2% agarose pad containing 2% galactose and 2% raffinose. Imaging was performed on a setup consisting of an AxioObserver inverted microscope (Zeiss), an alpha Plan-Apochromat 100× NA 1.46 oil objective, an sCMOS ORCA Flash 4v3 (Hamamatsu) with a 475–570 nm dichroic (Chroma), 570 nm longpass beamsplitter (Chroma) and 515/30 nm emission filter (Semrock), a UNO Top stage incubator (OKOlab) at 30°C, and LED excitation at 470/24 nm (SpectraX, Lumencor) at 20% power and an ND 2.0 filter, resulting in a 62 mW/cm^2^ excitation intensity. Widefield images were recorded for 1 h at 15 s interval, with *z*-stacks (9 planes at 0.5 μm intervals) and 150 ms exposure using Micro-Manager software (43). For each condition, 9 replicate datasets were acquired with in total at least 265 cells.

### Analysis of transcription dynamics

For analysis of the transcription dynamics imaging data, a similar approach was used as described previously (41). All analysis was implemented as custom-written Python software (10.5281/zenodo.7660780). First, images were corrected for *xy*-drift in the stage using an affine transformation on the maximum intensity projection. Next, cells were segmented using Otsu thresholding and watershedding. The intensity of the transcription sites (TS) was calculated by fitting a 2D Gaussian mask after local background subtractions as described previously (44). Initially, a threshold of eight times the standard deviation of the background was used. For frames where no TS was detected, a second fit was made in the vicinity of the high intensity spots detected in that cell, using a threshold of six times the standard deviation of the background. For frames where no TS was detected after this second fit, the intensity was measured at the location of the previous frame where a TS was successfully found. The tracking within each cell was inspected visually, and the endpoint of each trace was manually set at the last frame where a TS is visible. Cells without a TS, dividing cells, cells that were segmented incorrectly and cells that contained tracking errors were excluded from analysis.

To determine the on and off periods, binarization was performed using a threshold set at six times the standard deviation of the background. The standard deviation of the background was determined for each cell fitting by a Lorentzian distribution to intensities measured at four points at a fixed distance from the TS in each frame in the same cell. This threshold was chosen to reliably distinguish on and off periods from background levels at the single-transcript level. Subsequently, the binarization was improved by removing bursts that last a single frame and merging bursts that are separated by a single frame. From these binarized traces, the burst durations, time between bursts, induction time are directly calculated. The burst intensity is measured as the average intensity of all frames in which the cell was on. The fraction of active cells was determined by manual scoring of the cell that do and the cells that do not show a TS during the 1-h acquisition period.

In total at least 265 cells were included for each condition, and values for burst duration, time between bursts, induction time and burst intensity are determined by bootstrapping with 1000 repetitions. Reported error bars are standard deviations from the same bootstrap. Error bars in the number of active and inactive cells are given by the square root of the number of cells, as cells are expected to be independent of each other and thus follow Poisson statistics. To determine whether the obtained bursting parameters are significantly different between conditions, we have used bootstrap hypothesis testing using equation (4) from (45) to determine the achieved significance level.

### Protein detection by immunoblot and antibodies

Yeast cultures were started in synthetic complete medium containing the indicated carbon sources the morning, diluted in the evening and grown O/N to OD_600 nm_ 0.5, washed in MilliQ, pelleted and snap-frozen on dry ice. For protein extraction, cells were resuspended in 300 μl MilliQ, incubated with 300 μl 0.2M NaOH for 7 min at room temperature, centrifuged and resuspended in 500 μl 2× SDS-PAGE sample buffer (4% SDS, 20% glycerol, 0.1 M DTT, 0.125 M Tris–HCl pH 7.5 and EDTA-free protease inhibitors). Samples were incubated at 95°C for 5 min while shaking and centrifuged at 800g for 10 min at 4°C. A total of 20 μl lysate with loading buffer was run on a NuPAGE 3–8% gradient TAC gel and transferred to a 0.45-μm nitrocellulose membrane at 200 V, 1 A for 4 h at 4°C. For blocking, the membrane was washed with TBS-T, incubated with PBS containing 5% milk for 1 h and washed briefly with TBS-T, all at room temperature. The membrane was incubated with PBS containing 2% milk and primary antibody (1:5000) overnight at 4°C, washed three times with TBS-T for 10 min, incubated with 2% milk and secondary antibody (1:5000) for 1 h at room temperature, washed three times with TBS-T for 10 min and once with PBS for 10 min, and imaged using an LI-COR Odyssey IR imager (Biosciences). Western blot analysis was performed using primary antibodies against V5 (R960-25, ThermoFisher), Pgk1 (Invitrogen 22C5D8, RRID: AB_2532235) and tubulin (Ab6161, Abcam) and secondary antibodies Odyssey goat-anti-mouse 800 nm and Odyssey goat-anti-rat 800 nm.

The fluorescence signal of western blot images was quantified using ImageJ (46,47). In brief, ROIs of the same dimensions are drawn in each lane of the image. Next, a profile plot is created for each lane and a baseline is drawn manually to enclose the peak. The total area of the enclosed peak is calculated and used as a measure for the band intensity. This procedure is repeated for the signal of each primary antibody. The V5 band intensities are then normalized over corresponding Pgk1 or Tubulin bands and represented relative to the condition indicated in the figure legend.

### Growth assay

The galactose metabolism capacity of yeast strains was assessed with a growth assay as described previously (41), with minor modifications. Serial five-fold dilutions of indicated strains (YTL559, YTL1284, YTL1286, YTL1326 and YTL1098 in Figure 6A; BY4742 and YTL390 in Supplementary Figure S2A; YTL559, YTL390, YTL762 and YTL1304 in Supplementary Figure S3A; YTL559, YTL390, YTL1284, YTL945, YTL1226, YTL1221 and YTL1286 in Supplementary Figure S4A) were spotted on various plates and growth was assessed after 3 days at 30°C. Growth on YEP + 2% glucose was used as loading control. Growth on YEP + 2% galactose + 20 μg/ml ethidium bromide was interpreted as functional galactose metabolism, as galactose is the only carbon source available because ethidium bromide inhibits the use of amino acids as carbon source by binding to mitochondrial DNA. On the contrary, growth on YEP + 2% raffinose + 2% galactose + 40 mM lithium chloride (LiCl) + 0.003% methionine was interpreted as no functional galactose metabolism. Although galactose is present, its metabolism is lethal in the presence of LiCl due to the buildup of toxic metabolic intermediates. Therefore, only yeast without a functional galactose metabolism can survive on these plates, using the raffinose as carbon source. Methionine is added to prevent buildup of other toxic intermediates caused by LiCl inhibiting Hal2p/Met22p, the yeast BPNase (48).

### RT-qPCR

Yeast cultures were started in synthetic complete medium with 2% galactose and 2% raffinose in the afternoon and grown O/N to stationary phase. In the morning, cultures were diluted to OD_600 nm_ 0.125 and harvested at mid-log at OD_600 nm_ 0.5–0.6. Subsequently, cells were pelleted by centrifugation, snap-frozen on dry ice and stored at -80°C O/N. Three biological replicates were used for every yeast strain (BY4743, YTL1834, YTL1835, YTL1836 and YTL1837).

Total RNA was isolated using phenol-chloroform extraction. In brief, cells were resuspended in equal volumes (500 μl) of acid phenol:chloroform 5:1, pH 4.7 (Sigma) and TES buffer (10 mM Tris pH 7.5, 10 mM EDTA, 0.5% SDS), incubated at 65°C for 10 min followed by shaking at 1400 rpm at 65°C for 50 min. Aqueous and organic phases were separated by centrifugation at 14 000 rpm at 4°C for 20 min. The aqueous phase was subsequently washed with equal volumes of acid phenol:chloroform 5:1, pH 4.7 (Sigma) and phenol:chloroform:isoamyl alcohol 25:24:1 (saturated with 10 mM Tris, pH 8.0, 1 mM EDTA; Sigma) followed by 20 s of vortexing and phase separation by centrifugation at 14 000 rpm at 4°C for 10 min. The aqueous phase was then added to 50 μl sodium acetate (3 M, pH 5.2), diluted with cold (−20°C) 100% ethanol and left at −20°C for at least 30 min for the RNA to precipitate. RNA was pelleted by centrifugation at 14 000 rpm for 5 min after which the pellet was washed with 500 μl cold (−20°C) 80% ethanol, resuspended in water, snap-frozen in liquid nitrogen and stored at -80°C O/N. The isolated RNA was then cleaned using the RNeasy Mini Kit (QIAGEN) with on-column DNase treatment (QIAGEN) according to manufacturer's instructions. Per sample, 2500 ng RNA, measured using NanoDrop, was reverse transcribed using Tetro Reverse Transcriptase (Bioline) and Oligo(dT)18 primers (Meridian Bioscience), including a no-RT control reaction. For both reactions, a test PCR (same reaction as qPCR) with *ACT1* primers was performed and assessed by gel electrophoresis which verified the absence of PCR product in the no-RT control and a single band in RT reaction. The resulting cDNA was stored at −20°C.

RT-qPCR was performed in triplicates with 0.6 ng cDNA in 10 μl reactions using the SensiFAST No-ROX mix (Bioline #98020) and run on a LightCycler 480 System (Roche) using the following thermocycling parameters: 95°C for 5 min, 45 cycles (95°C for 10 s, 56°C for 10 s, 72°C for 10 s), followed by a dissociation curve. Amplicon lengths ranged from 150 to 179 bp. For every primer pair, eight five-fold dilutions (15 to 0.000192 ng) of BY4743 cDNA were included which confirmed that the obtained *C*_q_ values fell within the linear amplification range (*R*^2^ standard curves > 0.997). The Δ*C*_q_ values of the relative mRNA expression levels of *GAL1*, *GAL10*, *GAL7* and *GAL2* against *ACT1* were normalized to the average Δ*C*_q_ of the three biological replicates of WT/WT (BY4743). Significance between the Δ*C*_q_ values was determined using Student's *t* test.

## RESULTS

### Gal4 forms clusters in living yeast cells

To visualize endogenous Gal4 in living yeast cells, we fused EGFP with a flexible linker to the C-terminus of Gal4 (Figure 1A). Addition of the EGFP tag did not affect cell growth on galactose-containing plates, indicating full functionality in inducing the *GAL* genes (Supplementary Figure S2A). *GAL4-EGFP* cells were grown in media with different carbon sources, where the *GAL* genes are either repressed (glucose), uninduced (raffinose) or induced (galactose) (49), and imaged in 3D with high signal-to-noise ratio using highly inclined and laminated optical sheet (HILO) microscopy (50). In all three growth conditions, we observed bright Gal4-EGFP foci of high local concentration (Figure 1B), hereafter referred to as Gal4 clusters. These clusters were not observed in the WT strain (without *EGFP*) or a strain expressing nuclear *EGFP* (Supplementary Figure S2B), indicating that the observed clustering is specific for Gal4. To quantify the size and intensity of the observed clusters, they were fit with a 3D gaussian model. This allowed extraction of the peak width (the standard deviation *σ*, a measure for cluster size) and peak height (cluster density, a measure for Gal4 concentration within the cluster), which together determine the total intensity (the integrated peak intensity, a measure for the total number of molecules in the cluster) (Supplementary Figure S1A). Fitting of beads confirmed the independence of the size and density in the fitting algorithm and allowed us to use *σ* to estimate the cluster diameter (Supplementary Figure S1B–D). Quantification and localization of the clusters with this algorithm revealed that both the number of Gal4 clusters and the total cluster intensity were lowest in repressed cells (glucose), intermediate in uninduced cells (raffinose) and highest in induced cells (galactose) (Figure 1C, F). Repressed cells showed less dense clusters than uninduced and induced cells, while induced cells showed larger clusters than repressed or uninduced cells (Figure 1D, E). In induced conditions, the cluster diameter ranged approximately between 100 and 750 nm (Figure 1D, Supplementary Figure S1F, see Materials and Methods). We conclude that the TF Gal4 forms clusters and that the degree of clustering positively correlates with conditions of active *GAL* gene transcription.

### Gal4 clusters are enriched at their endogenous target genes

To test whether these Gal4 clusters localize at endogenous target sites, we integrated 128 repeats of the *tetO* sequence downstream of the *GAL1* gene and constitutively expressed tetR-tdTomato to visualize the location of the *GAL1–GAL10–GAL7* locus inside living cells (Figure 1G) (39). The *GAL* locus contains six Gal4 binding sites: four in the *GAL1-10* promoter and two in the *GAL7* promoter. Dual-color imaging of Gal4-EGFP clusters and the *GAL* DNA label in uninduced and induced conditions revealed frequent colocalization of Gal4 clusters at the *GAL* genes (Figure 1H). In both conditions, the Gal4 clusters with the highest density were found in proximity (3D distance < 400 nm) of the *GAL* locus (Figure 1I). These close-proximity clusters were, however, slightly smaller than clusters further away from the locus (Supplementary Figure S2F), such that the total intensity was increased but not as much as the density (Supplementary Figure S2G, H). Clustering around the multiple binding sites in the *GAL* locus may thus result in more concentrated, smaller Gal4 clusters.

To quantify the colocalization of clusters with the *GAL* locus, we determined the 3D nearest neighbor distance (NND) from each DNA label to a Gal4 cluster, which showed a clear peak at proximal distances (Figure 1J). To discriminate clusters that overlap with the *GAL* locus, the percent overlap was calculated using different distance thresholds (Supplementary Figure S2D). We chose a threshold of 400 nm to define overlapping clusters, since this threshold included the majority of the proximal high-intense clusters in uninduced conditions, while simultaneously limiting random overlap within the small yeast nucleus (<2 μm diameter). We note that the NND distribution is wider in induced than in uninduced condition (Figure 1J), perhaps because the *GAL* locus expands upon transcription activation, and that the 400 nm threshold likely results in an underestimate of the true overlap. Throughout the manuscript, we ensured that the results are independent of the chosen threshold. Using a threshold of 400 nm, 34 ± 3% and 60 ± 4% of the *GAL* loci overlap with a Gal4 cluster in uninduced and induced cells, respectively (Figure 1J, inset).

To check whether this colocalization was specific for the *GAL* genes, the DNA label was also placed in a different yeast strain downstream (3′) of *RNR2*, a gene located on a chromosome without any *GAL* genes and that transcribes independently of Gal4 binding (41) (Figure 1G, J). In contrast to the *GAL* DNA label, the Gal4 clusters with the highest density were not enriched at the *RNR2* DNA label (Supplementary Figure S2G). In addition, compared to the *GAL* genes, the distribution of NNDs at *RNR2* was much broader (Figure 1J) and only showed 10 ± 2% and 19 ± 3% overlap in uninduced and induced conditions, respectively. A second version of the *RNR2* DNA label, in which the label was placed upstream (5′) of *RNR2*, showed similar results (Supplementary Figure S2E-H). To confirm these results and to eliminate a possible bias from the lower *z*-resolution compared to the *x-y* resolution, we repeated this analysis in 2D (*xy*), which also showed many more proximal Gal4 clusters at the *GAL* genes than at the *RNR2* gene (Supplementary Figure S2I). These results indicate that Gal4 clusters are enriched at the *GAL* locus in both uninduced and induced conditions.

### The inhibitor Gal80 limits Gal4 cluster formation

**Figure 2.**
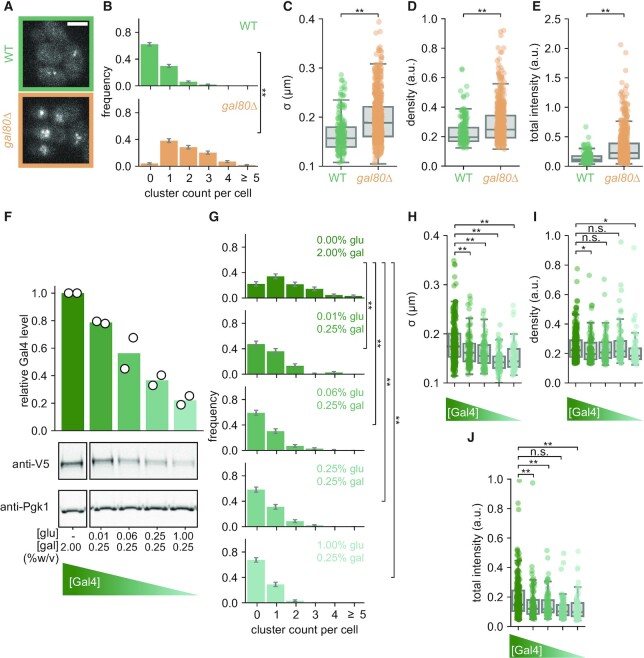
Gal4 cluster formation is inhibited by Gal80 and dependent on Gal4 concentration. (**A**) Representative images of Gal4-EGFP clusters in WT (green) and *gal80Δ* (orange) yeast cells in uninduced (raffinose) conditions. Images are a single *z*-slice of a representative group of cells. Scalebar: 3 μm. (B–E) Quantification of Gal4-EGFP clusters in WT (green, 357 cells) and *gal80Δ* (orange, 285 cells) in uninduced (raffinose) conditions. (**B**) Distribution of number of clusters observed per cell. Error bars indicate SEM based on 1000 bootstrap repeats. (C–E) Distribution of (**C**) cluster *σ*, (**D**) density and (**E**) total intensity. Circles show data for individual clusters and box plots show the distribution of the data, with box edges indicating first and third quartiles, center line indicating the median and whiskers indicating the 1.5× interquartile range. Significance determined by Mann–Whitney *U* test; **P < 0.01. (**F**) Western blot quantification of Gal4-EGFP-V5 protein levels using an anti-V5 antibody measured in the *gal80Δ* background across a range of glucose and galactose concentrations (%w/v) as indicated. Expression levels are normalized to Pgk1 and to the expression level in 2% galactose in the absence of glucose. Open circles represent the results of individual replicate experiments, bars indicate their mean. Western blot images are a representative example of two independent experiments. (G–J) Quantification of Gal4-EGFP clusters in *gal80Δ* background in the same glucose and galactose conditions as in (F), with in total 153, 114, 142, 151 and 179 cells included in the respective conditions. (**G**) Distribution of number of clusters observed per cell. Error bars indicate SEM based on 1000 bootstrap repeats. (H–J) Distribution of (**H**) cluster *σ*, (**I**) density and (**J**) total intensity. Circles show data for individual clusters and box plots show the distribution of the data, with box edges indicating first and third quartiles, center line indicating the median and whiskers indicating the 1.5× interquartile range. Significance determined by Mann–Whitney *U* test; n.s.: not significant; *P < 0.05; **P < 0.01.

As Gal4 clustering differs between growth conditions, we investigated how Gal4 clustering is regulated. We focused on regulatory features that differ between different sugar conditions, including Gal80-mediated inhibition of Gal4 activity, Gal4 interactions with the transcriptional machinery and Gal4 expression levels. In repressed and uninduced conditions (glucose and raffinose), Gal80 inhibits Gal4 activity. To test whether Gal80 inhibition limits Gal4 clustering in uninduced conditions, we analyzed Gal4 clustering in a *gal80Δ* strain in raffinose (Figure 2A). Western blot analysis of Gal4-EGFP-V5 verified that the Gal4 expression levels in *gal80Δ* cells were comparable to WT *GAL80* cells (Supplementary Figure S3B). Deletion of *GAL80* increased Gal4 cluster abundance, cluster size, cluster density and total cluster intensity (Figure 2B–E), indicating that Gal80 limits the clustering capability of Gal4 in uninduced conditions.

### Gal4 interactions with the Mediator tail are not required for Gal4 clustering

When Gal80 inhibition is relieved, Gal4 interacts with Mediator via dynamic ‘fuzzy’ interactions with the Med15 subunit (15) (Supplementary Figure S5A). Med15 forms condensates *in vivo* in mammalian cells (51), and Mediator condensates are able to induce liquid-liquid phase separation of ADs of various TFs *in vitro* (4). Because Gal80 shields the Gal4 AD and limits Gal4 clustering, we tested whether interactions of Gal4 with Med15 are important for Gal4 clustering. Comparison of Gal4 clusters between WT and *med15Δ* cells revealed that clusters were slightly more abundant, denser, and larger and had a slightly higher total intensity upon Med15 deletion (Supplementary Figure S3C–F). These results indicate that interactions with Med15 are not required for Gal4 clustering and may even limit them. Inhibition of Gal4 cluster formation by Gal80 thus occurs independent of suppression of Gal4-Mediator interactions. Gal80 may instead suppress Gal4–Gal4 self-interactions by physically shielding the AD or by enabling a more structured conformation of the AD (15).

### Gal4 clustering is concentration-dependent

In addition to Gal80-mediated regulation of Gal4 activity, *GAL4* expression is repressed by Mig1 in the presence of glucose (36). In line with previous studies, cells grown in repressed conditions showed reduced Gal4-EGFP-V5 levels by western blot compared to uninduced and induced conditions (Supplementary Figure S2C). Surprisingly, however, Gal4-EGFP-V5 levels were higher in cells grown in galactose than in raffinose, even though galactose was previously shown not to increase Gal4 mRNA levels, nor increase expression of a reporter gene driven by the *GAL4* promoter (52,53). We speculate that the Gal4 protein level increase in galactose may be caused by changes in its protein degradation rate. Regardless, these differences in Gal4 protein levels across these sugar conditions raised the question whether Gal4 clustering is concentration dependent, as has been found for other TFs (4,30,34).

To test the relationship between Gal4 concentration and Gal4 clustering, we varied the Gal4 concentration by modulating the glucose concentration (Figure 2F) (36) and analyzed Gal4 clustering at these varying Gal4 protein levels (Figure 2G–J). These latter experiments were performed in *gal80Δ* cells to prevent confounding effects of Gal80-regulation on Gal4 clusters. At higher Gal4 protein levels, more and larger clusters were observed, with minor differences in cluster density (Figure 2G–J). Overall, we conclude that Gal4 clusters are regulated by Gal80 and are concentration dependent.

### DNA binding facilitates, but is not essential for Gal4 clustering

**Figure 3.**
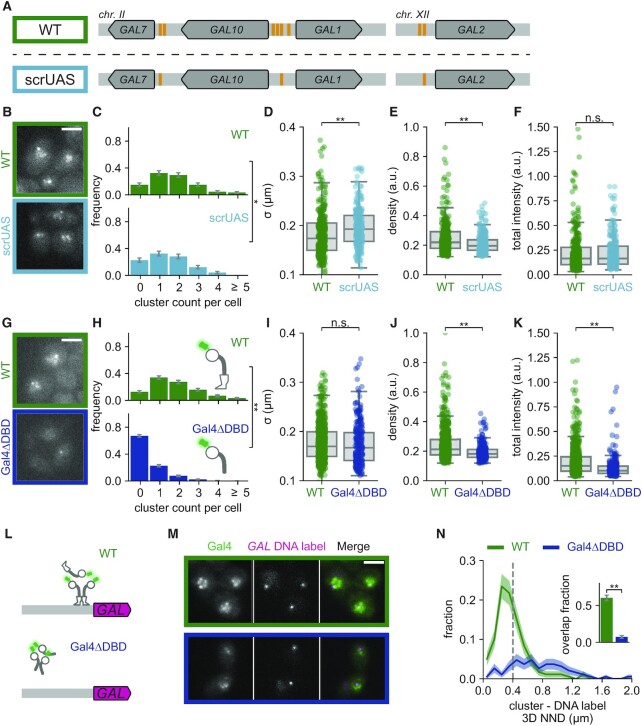
DNA binding is not essential for Gal4 clustering but is essential for cluster localization. (**A**) Schematic representation of the *GAL* genes with multiple UAS sites (orange) in WT (top, green box) and in a mutant with scrambled UAS sites (scrUAS; bottom, cyan box) such that only a single UAS site remains present in the promoter of each *GAL* gene (*scrUAS 5*′*GAL2* + *scrUAS 5*′*GAL7* + 3x *scrUAS 5*′*GAL10*, bottom, cyan box). (**B**) Representative images of Gal4-EGFP clusters in WT (green) and scrUAS (cyan) yeast cells in induced (galactose and raffinose) conditions. Images are a single *z*-slice of a representative group of cells. Scalebar: 3 μm. (C–F) Quantification of Gal4-EGFP clusters in WT (green, 173 cells) and scrUAS (orange, 163 cells) in induced (galactose and raffinose) conditions. (**C**) Distribution of number of clusters observed per cell. Error bars indicate SEM based on 1000 bootstrap repeats. (D–F) Distribution of (**D**) cluster *σ*, (**E**) density and (**F**) total intensity. Circles show data for individual clusters and box plots show the distribution of the data, with box edges indicating first and third quartiles, center line indicating the median and whiskers indicating the 1.5× interquartile range. Significance determined by Mann–Whitney *U* test; n.s.: not significant; *P < 0.05, **P < 0.01. (**G**) Representative images of WT (Gal4-EGFP; green) and Gal4ΔDBD (BPSV40-Gal4(Δ1–94)-EGFP; blue) clusters in yeast cells in induced (galactose and raffinose) conditions. Images are a single *z*-slice of a representative group of cells. Scalebar: 3 μm. (**H–K**) Same as (C–F) for WT Gal4 (Gal4-EGFP; green, 313 cells) and Gal4*Δ*DBD (BPSV40-Gal4(Δ1–94)-EGFP; blue, 517 cells) clusters in induced conditions. Significance determined by Mann–Whitney *U* test; n.s.: not significant; **P < 0.01. (**L**) Schematic representation of the *GAL* locus with DNA label (magenta) and clusters of either WT (Gal4-EGFP) or Gal4ΔDBD (Gal4ΔDBD-EGFP). (**M**) Representative images of dual-color fluorescence imaging to determine the colocalization between clusters (green) of either WT (green outline) or Gal4ΔDBD (blue outline) and the *GAL* locus (magenta) in induced conditions. Images are a single *z*-slice of a representative group of cells. Scalebar: 3 μm. (**N**) Distribution of 3D nearest neighbor distances (NND) between the *GAL* DNA label and the closest cluster of WT Gal4 (green, 200 cells) and Gal4ΔDBD (blue, 211 cells). Shaded regions represent SEM based on 1000 bootstrap repeats. Vertical dashed line indicates 400 nm threshold used to discriminate between overlapping and non-overlapping clusters. Inset shows fraction of *GAL* loci with an overlapping cluster. Error bars represent SEM based on 1000 bootstrap repeats. Significance determined by Fisher's exact test; **P < 0.01.

Next, we examined how Gal4 clustering is affected by DNA binding. The upstream activating sequences for galactose (UASg) contain one or more 17-bp 5′-CGG-N11-CCG-3′ consensus sequences, to which Gal4 binds as a dimer (54,55). Since the UASg of several Gal4 target genes contain multiple Gal4 upstream activating sequences (UASs): 4 UASs in *pGAL1-10*, 2 UASs in *pGAL7* and 2 UASs in *pGAL2* (Figure 3A), clusters could possibly arise from binding of several Gal4 molecules to the adjacent UASs in these promoters. We tested this by scrambling all-but-one UAS for each of these three target promoters, such that the consensus sequence is lost (scrUAS, Figure 3A) and no adjacent Gal4 bindings sites are left in the genome (56). In this scrUAS strain, Gal4 clusters did not disappear and were only mildly affected (Figure 3B–F), indicating that Gal4 clusters do not simply reflect multiple Gal4 molecules that are bound to adjacent binding sites. When adjacent UASs were scrambled, cluster abundance was only slightly reduced compared to a WT UAS strain, but interestingly, the clusters became larger and less dense (Figure 3C–E). Binding to adjacent sites, in particular at the *GAL1–GAL10–GAL7* locus, may thus enhance cluster concentration and reduce their size.

To investigate further how DNA binding affects Gal4 clustering, we deleted the N-terminal region containing both the DNA binding domain (DBD) and the dimerization domain (55,57,58). As this region also contains the nuclear localization signal (NLS) (59,60), nuclear localization was ensured by addition of a strong BPSV40 NLS (61). As expected, the Gal4ΔDBD mutant strain was unable to grow on galactose-containing plates (Supplementary Figure S4A). We found that deletion of the DBD did not abolish clustering but decreased cluster abundance, density and total intensity (Figure 3G–K). This reduction in clustering was not caused by lower Gal4 levels, as V5-tagged Gal4ΔDBD levels by western blot were modestly increased compared to Gal4 WT (Supplementary Figure S4B). In addition, to distinguish between the effect of DNA binding and dimerization, we mutated a single amino acid, S41D, which abolishes Gal4 binding to the endogenous *GAL1–10* promoter *in vivo* and to DNA *in vitro* but still contains an intact dimerization domain (62). Similar to Gal4ΔDBD, Gal4(S41D) clusters were less abundant and less dense than WT (Supplementary Figure S4D–G). DNA binding thus contributes to cluster formation and allows for more concentrated clusters, but it is not essential for clustering.

Analysis of Gal4ΔDBD cluster localization using the *GAL* DNA label showed that deletion of the DNA binding domain resulted in loss of cluster enrichment (7 ± 2% overlap) at the *GAL* locus (Figure 3L–N, Supplementary Figure S4H), in line with the essential function of DNA binding domains in determining sequence-specificity. Together, these results indicate that in the absence of DNA binding clusters can no longer localize at target genes, but can still form, albeit at a much-reduced rate.

### IDRs are not essential for Gal4 clustering, but contribute to target search

Our results show that the DBD of Gal4 is essential for cluster localization to the *GAL* genes, which is consistent with the sequence-specificity of DBDs. In addition, several reports have indicated that TF target gene selection is facilitated by regions outside DBDs (24,63,64). In some cases, the DBD is even dispensable for target search (24,65). For Gal4, the central region (CR) and C-terminal AD enhance localization of the DBD to an *in vivo* reporter array (66). Both these regions have been predicted and shown to contain disordered regions (15,67,68) (Supplementary Figure S5A). Since IDRs have previously been linked to cluster formation and target search (13,24,25), we wondered how the CR and AD of Gal4 contribute to cluster formation and localization.

**Figure 4.**
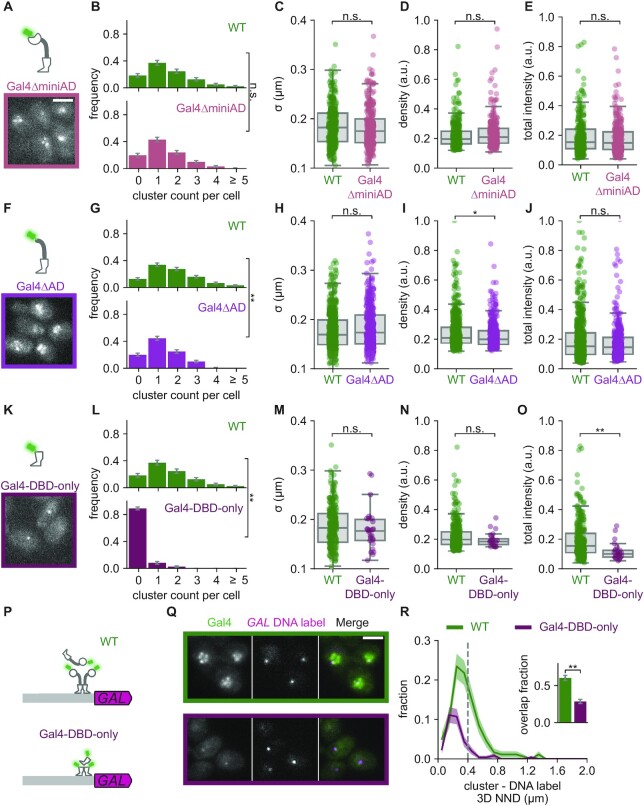
IDRs are not essential for Gal4 clustering but contribute to target search. (**A**) Top: Schematic representation of Gal4ΔminiAD (Gal4(Δ840–881)-EGFP). Bottom: Representative image of Gal4ΔminiAD clusters in yeast cells in induced (galactose and raffinose) conditions. Image is a single *z*-slice of a representative group of cells. (B–E) Quantification of WT Gal4 (Gal4-EGFP; green, 175 cells) and Gal4ΔminiAD (pink, 175 cells) clusters in induced conditions. (**B**) Distribution of number of clusters observed per cell. Error bars indicate SEM based on 1000 bootstrap repeats. (C–E) Distribution of (**C**) cluster *σ*, (**D**) density and **E**. total intensity. Circles show data for individual clusters and box plots show the distribution of the data, with box edges indicating first and third quartiles, center line indicating the median and whiskers indicating the 1.5× interquartile range. Significance determined by Mann–Whitney *U* test; n.s.: not significant. (**F**) Top: Schematic representation of Gal4ΔAD (Gal4(Δ768–881)-EGFP). Bottom: Representative image of Gal4ΔAD clusters in yeast cells in induced conditions. Image is a single *z*-slice of a representative group of cells. (**G–J**) Same as (B–E**)** for WT Gal4 (green, 313 cells) and Gal4ΔAD (magenta, 270 cells) clusters in induced conditions. Significance determined by Mann–Whitney *U* test; n.s.: not significant; *P < 0.05; **P < 0.01. (**K**) Top: Schematic representation of Gal4-DBD-only (Gal4(Δ95–881)-EGFP). Bottom: Representative image of Gal4-DBD-only clusters in yeast cells in induced conditions. Image is a single *z*-slice of a representative group of cells. (**L–O**) Same as (B–E) for WT Gal4 (green, 175 cells) and Gal4-DBD-only (purple, 193 cells) clusters in induced conditions. Significance determined by Mann–Whitney *U* test; n.s.: not significant; **P < 0.01. (**P**) Schematic representation of the *GAL* locus with DNA label (magenta) and clusters of either WT Gal4 (green) or Gal4-DBD-only (purple). (**Q**) Representative images of dual-color fluorescence imaging to determine the colocalization between clusters (green) of either WT Gal4 (green outline) or Gal4-DBD-only (purple outline) and the *GAL* DNA label (magenta) in induced conditions. Images are a single *z*-slice of a representative group of cells. Scalebar: 3 μm. (**R**) Distribution of 3D nearest neighbor distances (NND) between the *GAL* DNA label and the closest cluster of WT Gal4 (green, 200 cells) and Gal4-DBD-only (purple, 276 cells). Shaded regions represent SEM based on 1000 bootstrap repeats. Vertical dashed line indicates 400 nm threshold used to discriminate between overlapping and non-overlapping clusters. Inset shows fraction of DNA label-containing cells with an overlapping cluster. Error bars represent SEM based on 1000 bootstrap repeats. Significance determined by Fisher's exact test; ** *P* < 0.01.

To address this, we constructed three truncation mutants: I) Gal4ΔminiAD, lacking the last 40 amino acids of the AD (Supplementary Figure S5A); II) Gal4ΔAD, lacking the entire disordered AD; and III) Gal4-DBD-only, lacking the CR and the AD, and thus consisting only of the DBD and dimerization domain. All Gal4 truncation mutants showed higher Gal4 protein levels compared to WT Gal4 (Supplementary Figure S4B, C), and were unable to grow on galactose (Supplementary Figure S4A). The Gal4ΔminiAD and Gal4ΔAD showed similar or slightly fewer clusters of similar size and density compared to WT (Figure 4A–J), indicating that interactions between the AD and the transcriptional machinery do not have a major effect on clustering. Unexpectedly, the Gal4-DBD-only still formed clusters, despite lacking most, if not all, of the IDRs (Figure 4K). However, clusters were severely reduced in number and total intensity compared to WT Gal4 (Figure 4L–O). These remaining clusters cannot be explained as binding of multiple Gal4-DBD-only dimers to adjacent UASs, since these Gal4-DBD-only clusters still persisted when all adjacent UASs were scrambled (Supplementary Figure S5B-E). Together, these truncation experiments demonstrate that Gal4 clustering is mediated by multiple protein domains and that IDRs contribute to, but are not essential for Gal4 clustering.

The large reduction of clustering in the Gal4-DBD-only mutant suggests that the CR and AD contribute to self-interactions that enable cluster formation. We next asked whether these CR and AD-mediated self-interactions contribute to localization of clusters to target genes. To test this, we measured the overlap of the Gal4-DBD-only clusters with the endogenous *GAL* gene locus and found 29 ± 3% overlap (Figure 4P–R). Importantly, in the Gal4-DBD-only mutant many cells did not contain any clusters (Figure 4L, Supplementary Figure S5F), reducing the number of cells with a Gal4-DBD-only cluster at the *GAL* locus compared to WT (Figure 4R). The IDRs in the CR and AD thus contribute to the Gal4 cluster formation and localization at a target locus, in agreement with previous observations at artificial arrays (66).

### Gal4 self-interactions are sufficient to recruit Gal4 to target genes

**Figure 5.**
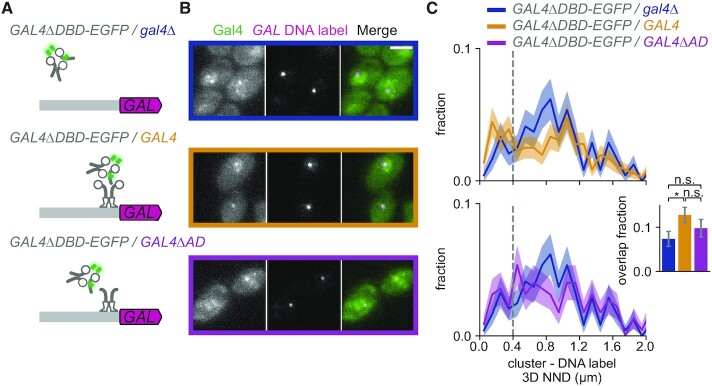
Gal4 self-interactions are sufficient to recruit Gal4 to target genes. (**A**) Schematic representation of the *GAL* locus with DNA label (magenta) and diploid yeast strains expressing Gal4ΔDBD-EGFP from one allele and on the other allele expressing either *gal4Δ* (*BPSV40-GAL4(Δ1–94)-EGFP/gal4Δ*, blue), WT Gal4 (*BPSV40-GAL4(Δ1–94)-EGFP/GAL4*, orange), or Gal4ΔAD (*BPSV40-GAL4(Δ1–94)-EGFP/GAL4(Δ768–881)*, purple). (**B**) Representative images of dual-color fluorescence imaging to determine the colocalization between Gal4ΔDBD-EGFP clusters (green) and the *GAL* DNA label (magenta) in the same yeast strains as (A) Cells were grown in uninduced conditions (raffinose) and imaged after 30 min of induction (galactose and raffinose). Images are a single *z*-slice of a representative group of cells. Scalebar: 3 μm. (**C**) Top: Distribution of 3D nearest neighbor distances (NND) between the *GAL* DNA label and the closest Gal4ΔDBD-EGFP cluster for *GAL4ΔDBD-EGFP/gal4Δ* (blue, 300 cells) and *GAL4ΔDBD-EGFP/GAL4* (orange, 423 cells). Bottom: same as top, for *Gal4ΔDBD-EGFP/gal4Δ* (blue, same dataset as top panel) and *GAL4ΔDBD-EGFP/GAL4ΔAD* (purple, 256 cells). Shaded regions represent SEM based on 1000 bootstrap repeats. Vertical dashed line indicates 400 nm threshold used to discriminate between overlapping and non-overlapping clusters. Inset shows fraction of DNA-label containing cells with an overlapping cluster. Error bars represent SEM based on 1000 bootstrap repeats. Significance determined by Fisher's exact test; n.s.: not significant; *P < 0.05.

Our mutant analysis indicated that although the CR and ADs of Gal4 facilitate Gal4 clustering (Figure 4), proper localization to the *GAL* locus requires a functional DBD (Figure 3). These findings suggested that the DBD may anchor Gal4 at the correct sites, and that CR- and AD-mediated self-interactions facilitate recruitment of additional Gal4 molecules and cluster formation. To test this model, we assessed whether the Gal4ΔDBD mutant could be recruited to the *GAL* locus through clustering with Gal4 molecules that do contain a DBD. This experiment was performed in a diploid yeast strain where one allele expressed Gal4ΔDBD-EGFP and the other allele contained either a *GAL4-*deletion (*gal4*Δ), full length WT Gal4 or Gal4ΔAD (containing the DBD and CR) (Figure 5A). As expected, in absence of an additional Gal4 copy, the Gal4ΔDBD clusters did not localize at the *GAL* locus (Figure 5A, B, top panel), and only 7 ± 2% of *GAL* DNA labels overlapped with a Gal4ΔDBD cluster (Figure 5B), which is the same as the overlap in haploid cells (7 ± 2%, Figure 3K). However, upon additional expression of WT Gal4, the Gal4ΔDBD-EGFP clusters showed a clear peak of enrichment at the *GAL* locus, and a significantly increased overlap with the *GAL* locus from 7 ± 2% to 13 ± 2% (Figure 5B, C, top panel). Expression of the Gal4ΔAD mutant also showed the same trend of increasing the localization of the Gal4ΔDBD-EGFP clusters to the *GAL* locus (10 ± 2%) (Figure 5B, C, bottom panel). This increase in overlap was independent of the chosen threshold to determine the overlap (Supplementary Figure S6A). In addition, analysis of the overlap in 2D instead of 3D revealed even clearer peaks of enrichment of Gal4 clusters close to the *GAL* genes in the presence of the Gal4ΔAD or Gal4 WT, but not when the second allele was deleted (Supplementary Figure S6B). These results indicate that the Gal4ΔDBD mutant can be recruited to its target genes via protein-protein interactions with DBD-containing Gal4.

To independently validate these findings, we labeled the *GAL10* RNA with 14 repeats of PP7 hairpins to visualize the *GAL10* transcription site (TS). When the PP7 hairpin sequences at the 5′ of *GAL10* are transcribed, the nascent RNA is specifically bound by the PP7 coat protein, fused to a fluorescent protein (69,70). The *GAL10* TS is visible in the microscopy images as a bright nuclear spot. To measure recruitment of Gal4 to the active TS, we expressed either WT Gal4-EGFP or Gal4ΔDBD-EGFP from one allele and additional unlabeled Gal4 from a second allele to ensure transcriptional activity (Supplementary Figure S6C). For WT Gal4, around 75 ± 4% of *GAL10* TSs overlapped with a Gal4 cluster (Supplementary Figure S6D). This overlap is higher than the overlap with the *GAL* DNA label (60 ± 4%), which could hint at enrichment of colocalized Gal4 clusters when *GAL10* is active. Gal4 clusters that overlapped with the *GAL10* TS had a higher total intensity than Gal4 clusters that did not overlap (Supplementary Figure S6F), in agreement with our findings using the *GAL* DNA label (Supplementary Figure S2H). For Gal4ΔDBD-EGFP, a clear peak of close-proximity clusters was observed in the NND distribution, and approximately 42 ± 4% of *GAL10* TSs overlapped with a Gal4ΔDBD-EGFP cluster (Supplementary Figure S6D). These results are in line with our model that additional non-DNA-bound Gal4 molecules are recruited to target loci via protein-protein interactions, and that this recruitment is dependent on the Gal4 CR and/or AD. Transient self-interactions underlying Gal4 clusters are thus able to recruit Gal4 to its target genes.

### Colocalization of an active gene with a Gal4 cluster does not change transcriptional output

We next sought to understand how Gal4 clustering affects transcription activation. To link Gal4 clustering to the transcriptional output of its target genes, we used the PP7-*GAL10* RNA labeled strain to quantify transcription of the endogenous *GAL10* gene in living cells. In the microscopy images, the intensity of the TS is a measure for the number of nascent RNAs. To understand whether the colocalization of Gal4 is correlated to transcription levels, we compared the intensity of the *GAL10* TS in cells with and without an overlapping Gal4 cluster. This analysis showed that *GAL10* TSs that overlapped with a Gal4 cluster had the same intensity as non-overlapping TSs (Supplementary Figure S6E). The presence of a Gal4 cluster at a target gene is thus uncorrelated with the transcription level of the target gene.

### Gal4 self-interactions are insufficient to activate transcription

**Figure 6.**
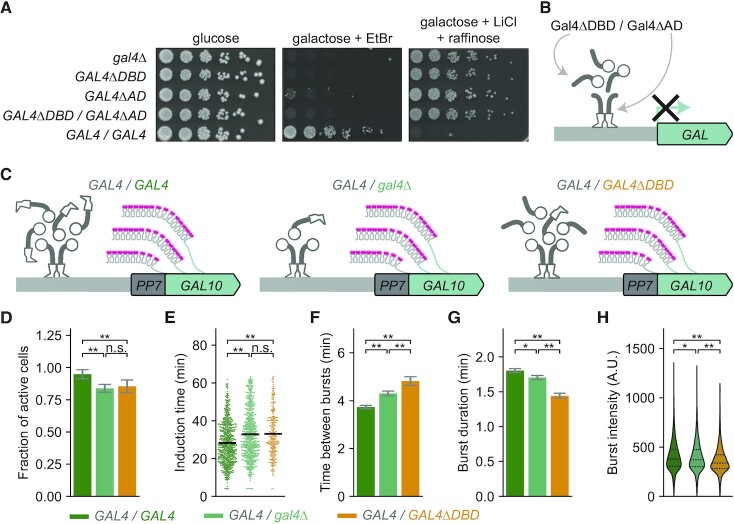
Gal4 self-interactions are insufficient to activate *GAL* gene transcription. (**A**) Growth assay of indicated Gal4-EGFP mutants to assess their galactose metabolism capability. Shown are 5-fold serial dilutions on YEP + 2% glucose (dilution control), YEP + 2% galactose + 20 μg/ml ethidium bromide (growth = functional galactose metabolism) and YEP + 2% raffinose + 2% galactose + 40 mM lithium chloride + 0.003% methionine (no growth = functional galactose metabolism). (**B**) Schematic representation of impaired *GAL* gene transcription in a diploid yeast strain expressing Gal4ΔAD from one allele and Gal4ΔDBD from the other allele (*GAL4(Δ768–881)-EGFP/BPSV40-GAL4(Δ1–94)-EGFP*). (**C**) Schematic representation of the transcribed *PP7*-*GAL10* gene in diploid yeast strains expressing WT Gal4 from one allele and on the other allele expressing either WT Gal4 (*GAL4/GAL4*, green), no Gal4 (*GAL4/gal4Δ*, light green) or Gal4ΔDBD (*GAL4/GAL4ΔDBD*, orange*)*. Nascent *GAL10* transcripts are fluorescently labeled by binding of the PP7-coat protein-ymScarletI to the PP7-stem loops (magenta). (D–H) Quantification of *PP7-GAL10* transcription in yeast strains described in (C) with a total of 783, 889 and 350 cells, respectively. Cells were grown in uninduced conditions (raffinose) and imaged during 1 h of induction with galactose. (**D**) Fraction of cells that activate *PP7*-*GAL10* transcription within 1 h after induction. Error bars are propagated statistical errors in the number of active and inactive cells. Significance determined by Fisher's exact test; n.s.: not significant; **P < 0.01. (**E**) Distributions of induction times of *PP7*-*GAL10* transcription. Black horizontal line: average as determined by 1000 bootstrap repeats. (**F**) Average time between consecutive bursts and (**G**) average burst duration of *PP7*-*GAL10* transcription, in which average and errors are determined by 1000 bootstrap repeats. (**H**) Distribution of burst intensities of *PP7*-*GAL10* transcription. Black dashed lines show each quartile of the distribution. For (**F–H**), only cells with active *PP7-GAL10* transcription were included (673, 669 and 265 cells, respectively). Significance is determined by bootstrap hypothesis testing (45); n.s.: not significant; *P < 0.05; **P < 0.01.

The finding that Gal4 clustering does not correlate with the transcriptional activity of a target gene (Figures 1, 2, Supplementary Figure S6E) suggests that the Gal4 molecules in a cluster may not contribute to transcription activation. To test this further, we made use of the Gal4ΔAD and Gal4ΔDBD mutants that individually are unable to activate transcription as shown by their inability to grow on galactose-containing plates (Figure 6A). It is well-known that, when brought in close proximity, the Gal4 DBD and AD can function as transcriptional activator, even when they are part of different fusion proteins. This mechanism has been exploited in the classical Yeast-Two-Hybrid system to detect protein-protein interactions (71). Our experiments showed that the Gal4ΔAD is able to recruit the Gal4ΔDBD to the Gal4 locus (Figure 5). We reasoned that if clustered Gal4ΔDBD molecules contribute to transcription activation, transient interactions between the DBD of the Gal4ΔAD mutant and the AD of the Gal4ΔDBD mutant within a cluster should rescue their transcriptional inactivity. However, coexpression of Gal4ΔAD and Gal4ΔDBD in a diploid yeast strain neither rescued the ability to activate the *GAL* gene pathway, as evidenced by their inability to grow on galactose-containing plates (Figure 6A), nor activated transcription of individual *GAL* genes (Supplementary Figure S6G). Despite being localized at the *GAL* genes, the AD of the Gal4ΔDBD mutant was unable to activate transcription (Figure 6B), suggesting that transient self-interactions are insufficient to activate gene expression. Moreover, the lack of rescue suggests that non-DNA-bound molecules in a cluster do not activate transcription.

### Gal4 self-interactions at target genes can inhibit transcription

To further dissect the function of clustered non-DNA-bound Gal4 molecules in gene activation, we asked if clustered TF molecules that cannot bind DNA can enhance transcription activation in the presence of WT Gal4. We compared *GAL10* transcription between three diploid strains, all expressing WT Gal4 to activate transcription, and on the other allele either *GAL4*, *gal4Δ* or *GAL4ΔDBD* (Figure 6C). To quantify transcriptional activity, *GAL10* transcription was measured in live cells by PP7-*GAL10* imaging directly upon galactose addition. Compared to the cells with two *GAL4* gene copies (*GAL4/GAL4*), cells with one *GAL4* gene copy (*GAL4/gal4Δ*) had a slightly lower active fraction and took a longer time to activate *GAL10* after induction with galactose. Additionally, transcriptional bursts were shorter and time between consecutive bursts was longer (Figure 6D–H, Supplementary Figure S6H-J). If clustering of non-DNA-bound Gal4 molecules at a target site contributes to target gene transcription, nascent *GAL10* transcription in cells expressing Gal4ΔDBD on the second allele (*GAL4/GAL4ΔDBD)* should be increased compared to cells with only one Gal4 copy (*GAL4/gal4Δ)*. However, live-cell imaging revealed that the presence of the Gal4ΔDBD mutant decreased the transcriptional output of *GAL10* even further, as we observed even shorter transcriptional bursts and even longer time between consecutive bursts, as well as a decrease in the burst intensity (Figure 6D–H, Supplementary Figure S6H–J). Therefore, we conclude that although the Gal4ΔDBD molecules can be recruited to a target site through self-interactions with WT Gal4 (Figure 5), these molecules do not contribute to transcription activation, and even inhibit gene transcription (Figure 6). Overall, our results indicate that the self-interactions that mediate TF clustering facilitate TF localization to target genes, but may also negatively influence transcription by inhibiting gene activation.

## DISCUSSION

In this manuscript we used quantitative imaging in living cells to understand how clustering of the paradigm TF Gal4 is regulated and how it influences transcription factor function. We found that Gal4 cluster abundance, size and density are regulated in different growth conditions through at least two mechanisms: I) Glucose-repression of Gal4 expression levels decreased cluster abundance and size, and II) Gal80-mediated Gal4 inhibition decreased Gal4 cluster abundance, size and density. In contrast, Gal4 clustering is largely unaffected by the well-established interactions with the Mediator complex through the subunit Med15. Moreover, DNA binding enhances cluster formation, and enables clusters to become denser and smaller, especially at the multiple Gal4 UASs of the *GAL1-GAL10-GAL7* locus. Gal4 clustering is also facilitated by, but not completely dependent on the CR and AD, which contain several IDRs. Through combinations of various truncation mutants, we showed that clusters consist of DNA-bound Gal4 molecules as well as non-DNA bound Gal4 molecules that are likely recruited to target genes through protein-protein interactions of the CR and ADs. However, once at the target site, these non-DNA bound TF molecules do not contribute to gene activation, and may in fact inhibit gene transcription.

### Gal4 cluster formation

TF clusters have previously been described to be driven by both homotypic and heterotypic interactions (72). For Gal4, several findings suggest that clusters are formed mostly by homotypic rather than heterotypic interactions: I) Clustering was not reduced by loss of interactions with Med15. II) Although Med15 is only one of the heterotypic interaction partners, removal of the miniAD presumably results in loss of most heterotypic interactions with the transcriptional machinery (73), but yielded similar clustering as WT. III) Gal4 without a DBD can be recruited to the *GAL* locus by WT Gal4 or Gal4 without an AD. To show the contribution of homotypic self-interactions or heterotypic interactions of TFs with other factors, previous studies have used *in vitro* analysis with purified proteins (4,11,16). Gal4, however, has been notoriously difficult to purify as a full-length protein, preventing us from performing such *in vitro* experiments (74). Regardless, our imaging tools create the possibility to test in the future how Gal4 clustering is regulated by other Gal4 interactors *in vivo*, such as SAGA, TBP or TFIIB (73,75,76). Moreover, it will be interesting to explore whether such factors co-cluster with Gal4 near target genes, as was observed for Hsf1 condensates (29). Although the role of heterotypic interactions in Gal4 clustering remains unclear, our data suggests that homotypic self-interactions are an important contributor to Gal4 clustering.

Previously, homo- and heterotypic multivalent interactions have been linked to the formation of liquid-liquid phase separated condensates (4,72). The concentration-gradient experiment shows that increasing the Gal4 concentration increases the cluster abundance and size, but has minor effects on the cluster density (Figure 2). These results are consistent with the simplest liquid-liquid phase separation model, which postulates that at higher concentrations, more clusters are formed and that clusters merge into larger clusters with the same density inside the cluster (77,78). In this model, the nucleoplasmic Gal4 concentration is also expected to be stable, but the high number of clusters prevented us from measuring this background concentration reliably. Thus, although our concentration-gradient is consistent with this model, further tests would be required to understand the role of liquid-liquid phase separation in Gal4 cluster formation. In addition, the physical nature is of Gal4 clusters remains unclear, i.e. whether they represent liquid droplets, gels or other forms of biomolecular condensates (72). Current imaging settings using high excitation powers quickly resulted in bleaching, precluding us from studying Gal4 clusters for prolonged times. Future improvements of fluorescent proteins and microscopy systems may allow for longer imaging to determine the dynamics and nature of Gal4 clusters.

### Gal4 cluster regulation by Gal80

In inactive conditions, Gal4 clustering is limited by its inhibitor Gal80. Gal80 binds to the Gal4 AD, resulting in a structured conformation of the AD and masking it for interactions with transcription cofactors such as Mediator (79,80). Loss of Med15 did not majorly affect Gal4 clustering, indicating that the inhibitory effect of Gal80 on clustering is likely mediated by preventing homotypic interactions rather than by preventing Mediator interactions. Gal80 prevents these homotypic interactions likely by shielding a larger region than the AD, as Gal4 clusters also formed efficiently in the Gal4ΔAD mutant.

A previous study showed that Gal80 also localizes in nuclear clusters that dissociate upon galactose addition (81). Self-association of Gal80 was proposed to be required for Gal4 repression by Gal80, explaining why genes with multiple Gal4 UASs may be more efficiently repressed than genes with a single Gal4 UAS (82). The corollary of this model is that the inhibitory capacity of Gal80 may be enhanced by Gal4 clustering, but that Gal80 simultaneously inhibits Gal4 clustering, thereby limiting its own inhibitory capacity. Conversely, if Gal80 and Gal4 oligomerization enhances their function, perhaps the formation of clusters is merely a consequence of this function.

### Role of DNA binding and IDRs in Gal4 clustering

Clustering is often suggested to be related to IDR-mediated protein-protein interactions (4,12,17). However, the exact role of different TF domains remains unclear and may differ per TF. For example, for the pioneer factor Sox2 clustering depends on the DNA binding domain but not the activation domain (18), suggesting that clustering is the result of the spatial proximity of multiple TF binding sites. On the other hand, clustering of the Oct4 TF depends on the IDRs in the ADs (4). Here, we find that for Gal4, clustering depends both on DNA binding and the IDRs.

When the Gal4 DBD is deleted or mutated, clusters are still observed, although they are much less abundant and less dense (Figure 3). These effects on cluster abundance are larger compared to the UASscr mutant, indicating that DNA binding at single sites considerably contributes to cluster formation. However, the presence of multiple Gal4 UASs in individual promoters increases the cluster density. DNA binding may form a scaffold that lowers the propensity for cluster formation, which may be enhanced if multiple UASs are placed adjacently, similar to enhancers in mammalian cells (17,83). In addition, Gal4 cluster properties may be affected by the position of the *GAL* genes in the nucleus, as these genes are known to move to the nuclear periphery in induced conditions (84).

Besides DNA binding, Gal4 clustering is also facilitated by the IDRs in the CR and AD. Removal of all predicted IDRs, leaving only the structured DBD and dimerization domain of Gal4 (Gal4-DBD-only), resulted in a large decrease in cluster density and abundance (Figure 4). However, some clusters are still observed upon IDR loss, suggesting that the structured DBD and/or dimerization domain contribute to clustering. In accordance, clustering of IDRs was reported to be driven by their multivalency rather than by their disorderedness (85) and such multivalency may also be present in structured domains. Given that the dimerization domain is capable of interacting with a specific Med15 mutant (Gal11p) (58), we speculate that the dimerization domain may be able to form homotypic and heterotypic interactions that promote clustering. Along these same lines, besides the predicted IDRs, the CR also contains a predicted structured region (Supplementary Figure S5A) which may contribute to the clustering potential of the CR. We conclude that both DNA binding and IDRs contribute to Gal4 clustering.

### Self-interactions facilitate target search

In eukaryotic cells, TFs face a major challenge in finding their targets in millions of non-specific sequences. Our results indicate that self-interactions may facilitate this target search, in line with previous findings for several yeast and mammalian transcriptional regulators (24–28). Although localization of Gal4 clusters is dependent on the sequence-specific Gal4 DBD, self-interactions between regions outside the DBD promote the formation and localization of Gal4 clusters to the *GAL* locus. We envision that once Gal4 molecules are bound to the DNA, their exposed IDRs allow for interaction with additional unbound Gal4 molecules, thereby creating a larger effective target size. Such a mechanism may ensure that the DBD of each Gal4 molecule does not need to probe all DNA sequences to find its binding site, and reduces the search space to areas where other Gal4 molecules have already found their target (13,86).

In this model, clustering also creates cooperativity, as binding of subsequent molecules is enhanced after the first one is bound. It has been observed that Gal4 binds to the four binding sites in the *GAL1-10* promoter in a cooperative manner (87), and that this cooperativity depends on the Gal4 central region (88). Our observation that the central region is important for clustering (Figure 4) suggests that cooperativity and clustering are indeed correlated. Cooperative binding through clustering has also been proposed to be important for mammalian super-enhancers that contain many TF binding sites (22). For Gal4, four target genes contain multiple binding sites, which include the genes encoding galactose-metabolic enzymes (56). At these genes, cooperative binding from clustering may enable fast transcriptional response upon exposure to galactose and may ensure high Gal4 promoter occupancy and high target gene expression. Conversely, several other Gal4 target promoters only contain one UAS and it remains to be established whether clustering and self-interactions are beneficial for target search for these genes. Since a cluster located near a promoter locally increases the concentration of Gal4 molecules, we can imagine that, once a Gal4 molecule is released from the DNA, quick rebinding of another Gal4 from the nearby cluster to the DNA may increase the overall time that the promoter is occupied. In addition, self-interactions between the cluster and the bound Gal4 molecules could increase the dwell time of the bound molecule (12,21). It will be interesting in the future to examine how self-interactions and clustering influence Gal4 binding kinetics at different genes and at the single-molecule level.

### Clustered Gal4 molecules do not activate transcription

Although Gal4 clusters overlap with transcriptionally active loci, *GAL* gene transcription is not supported by additional non-DNA-bound Gal4 molecules in the cluster. In a yeast-two-hybrid-like setup, DNA-bound Gal4ΔAD without an AD that recruited a Gal4ΔDBD without a DBD to the target gene could not activate transcription (Figure 6). We speculate that the classical yeast-two-hybrid setup to detect protein-protein interaction only detects stable lock-and-key interactions and not ‘fuzzy’ interactions such as those facilitated by TF clustering. Moreover, the presence of extra non-DNA-bound mutant Gal4 molecules at the target gene inhibited rather than enhanced transcription activation. Based on these findings, we conclude Gal4 needs to be DNA-bound to activate transcription.

Our experiments with truncation mutants allowed us to establish that non-DNA bound molecules do not contribute to gene activation. We observe that these non-DNA bound molecules may inhibit transcription, but this could be due to a dominant-negative effect of the Gal4ΔDBD mutant. In these experiments, the concentration of DBDs within clusters is reduced, which may lower the on-rate compared to WT. Since such truncated proteins are not present in WT cells, we cannot directly test whether these results apply to WT clusters. However, given that transcription inhibition from clustering has been observed previously (33,34), it is conceivable that Gal4 clustering also inhibits gene expression in a WT context.

In this latter case, we speculate that the observed transcription inhibition inside clusters occurs through a titration mechanism called squelching (89). Gal4 overexpression has been described to inhibit transcription by titrating the transcriptional machinery (89). The high Gal4 concentration inside the cluster may cause competition between non-DNA-bound Gal4 with DNA-bound Gal4 for interactions with cofactors and the preinitiation complex. A similar transcription inhibition has recently been observed for the FUS-EWS fusion, where additional expression of the EWS domain decreased the ability of the endogenous FUS-EWS fusion to active transcription (33). Here, additional non-DNA-bound EWS may squelch DNA bound FUS-EWS. In addition, our model is in line with the recent finding that chemically-induced clustering caused a negative correlation between cluster intensity and transcriptional output (32). These findings suggests that self-interactions that cause clustering may compete with, rather than facilitate, recruitment of the transcriptional machinery. Future research is required to test whether squelching occurs inside clusters and to reveal which factors of the transcriptional machinery are titrated.

### Clustering acts as a double-edged sword

Transcription factors need to perform two major steps during transcription activation: binding to their target genes and activating transcription by recruiting the transcriptional machinery (1). Our results show that Gal4 clustering enables DNA binding but inhibits transcription activation. This finding suggests that cells need to balance these positive and negative aspects of clustering for proper gene expression. Depending on the propensity to engage in homotypic and heterotypic interactions, this balance may shift for different transcription factors. Interestingly, mutagenesis of the central AD of the yeast TF Gcn4 has revealed several mutations that increase cofactor recruitment and transcriptional activity (90), raising the question why increased heterotypic interactions are selected against in evolution. It has been suggested that weak ADs prevent squelching or allow for inducibility (90,91). It will be interesting to test if synthetic AD motifs with increased heterotypic interactions will come at the cost of homotypic interactions and target search. Such a balance may perhaps also clarify why clustering and liquid-liquid phase separation may be beneficial in certain systems and inhibitory in others. Our quantitative imaging approach will open up new avenues to explore this balance for other TFs in the future.

## DATA AVAILABILITY

Software for analysis and plotting of clustering microscopy data is available at Zenodo (10.5281/zenodo.7650154 with dependencies from 10.5281/zenodo.7650168 and 10.5281/zenodo.7650172). Software for analysis of transcription dynamics microscopy data is available at Zenodo (10.5281/zenodo.7660780). The results of spot and bead fitting, disorder predictions, growth assays, overlap fraction vs threshold, RT-qPCR, transcription dynamics and western blots as well as example images from clustering microscopy data and growth assays are available from Zenodo (10.5281/zenodo.7701752). All microscopy images are available from the corresponding author upon reasonable request.

## Supplementary Material

gkad227_Supplemental_File
