# Supplementary material for: Transcription factor clusters enable target search but do not contribute to target gene activation
Source: Nucleic Acids Res. 2023 Mar 29;51(11):5449–68. doi: 10.1093/nar/gkad227 (PMC10287935; doi:10.1093/nar/gkad227)
Supplement: gkad227_Supplemental_File [file gkad227_supplemental_file.pdf]

SUPPLEMENTARY DATA

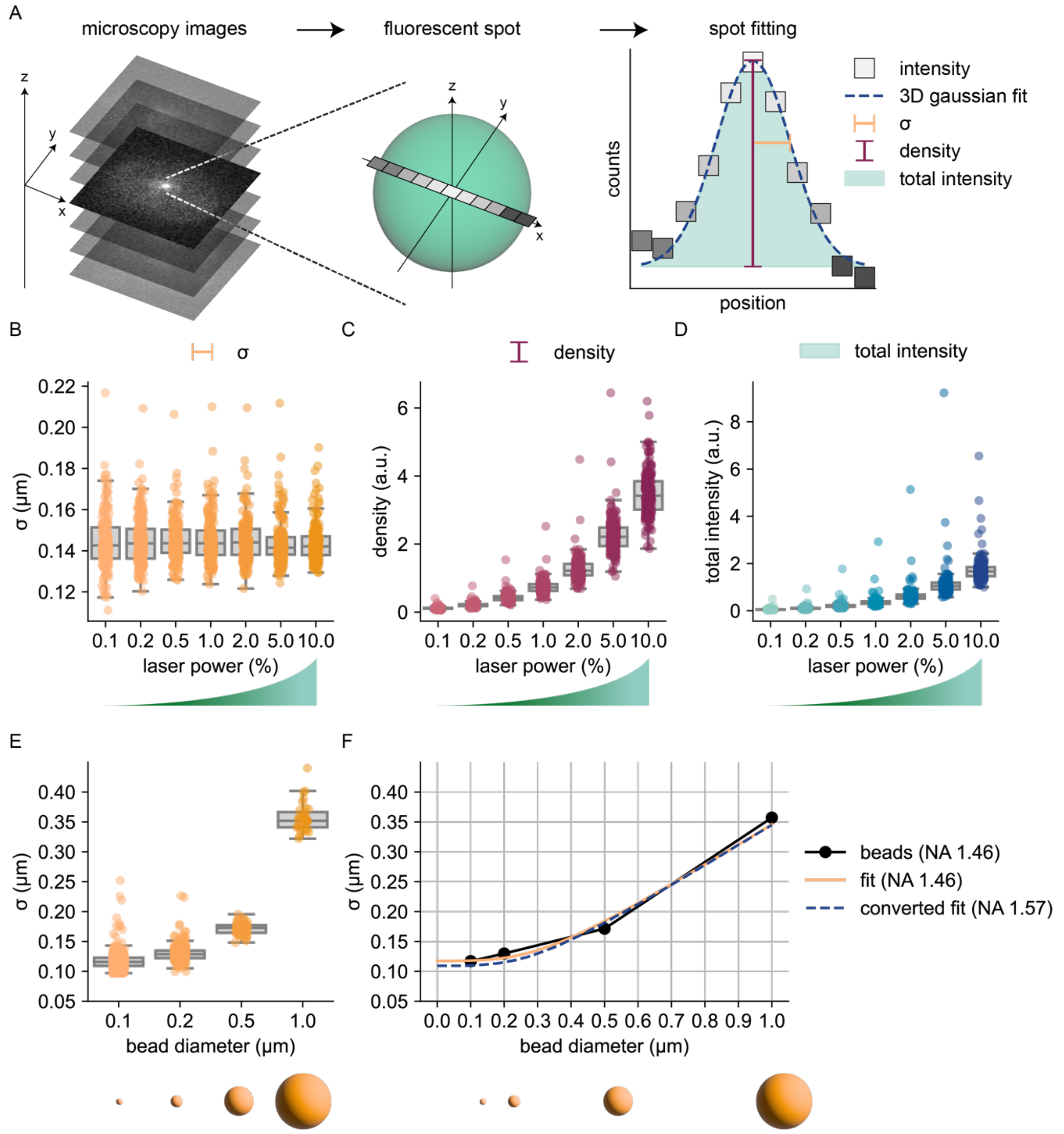

Figure S1. Spot fitting algorithm to extract the cluster σ, density and total intensity.

A. Schematic representation of data analysis, starting with a microscopy image of cells with fluorescent spots (clusters, DNA/RNA label), followed by a schematic of a detected spot with intensities measured at each pixel (grey squares). These intensities are fit to a 3D gaussian with background subtraction using a tilted plane, allowing extraction of σ (the standard deviation, a measure for cluster size), cluster density (peak height, a measure for concentration within the cluster) and the total integrated intensity (as a measure for the total number of molecules in the cluster).

B-D. Distribution of σ, density and total intensity of 202 individual fluorescent beads (0.21 µm TetraSpec microspheres) measured at different laser powers representing the width, peak height, and integrated intensity of the 3D gaussian fit, respectively (see methods for details). As expected, the spot density, but not the σ changes at different laser powers. Circles show data for individual beads and box plots show the distribution of the data, with box edges indicating first and third quartiles, center line indicating the median and whiskers indicating the 1.5x interquartile range.

E. Top: Distribution of σ of individual fluorescent beads (Tetraspec microspheres) with increasing diameters (see methods for details). For every bead diameter multiple *z*-stacks were taken, resulting in a total of 676, 310, 69 and 33 detected beads of respectively 0.1, 0.2, 0.5 and 1.0 µm in diameter. As expected, the σ changes for different bead sizes. Circles show data for individual beads and box plots show the distribution of the data, with box edges indicating first and third quartiles, center line indicating the median and whiskers indicating the 1.5x interquartile range. Bottom: schematic representation of increasing bead diameter (to scale).

F. Calibration curve to relate measured σ to spot diameter. Black: mean measured values for σ of the different diameter beads from E. Orange: fit to the black data taken with a NA 1.46 objective (see methods for details). Blue dashed line: conversion of fit curve to predicted relationship for NA 1.57 objective (as used for Gal4-EGFP clusters, see methods for details). Bottom: schematic representation of increasing bead diameter (to scale).

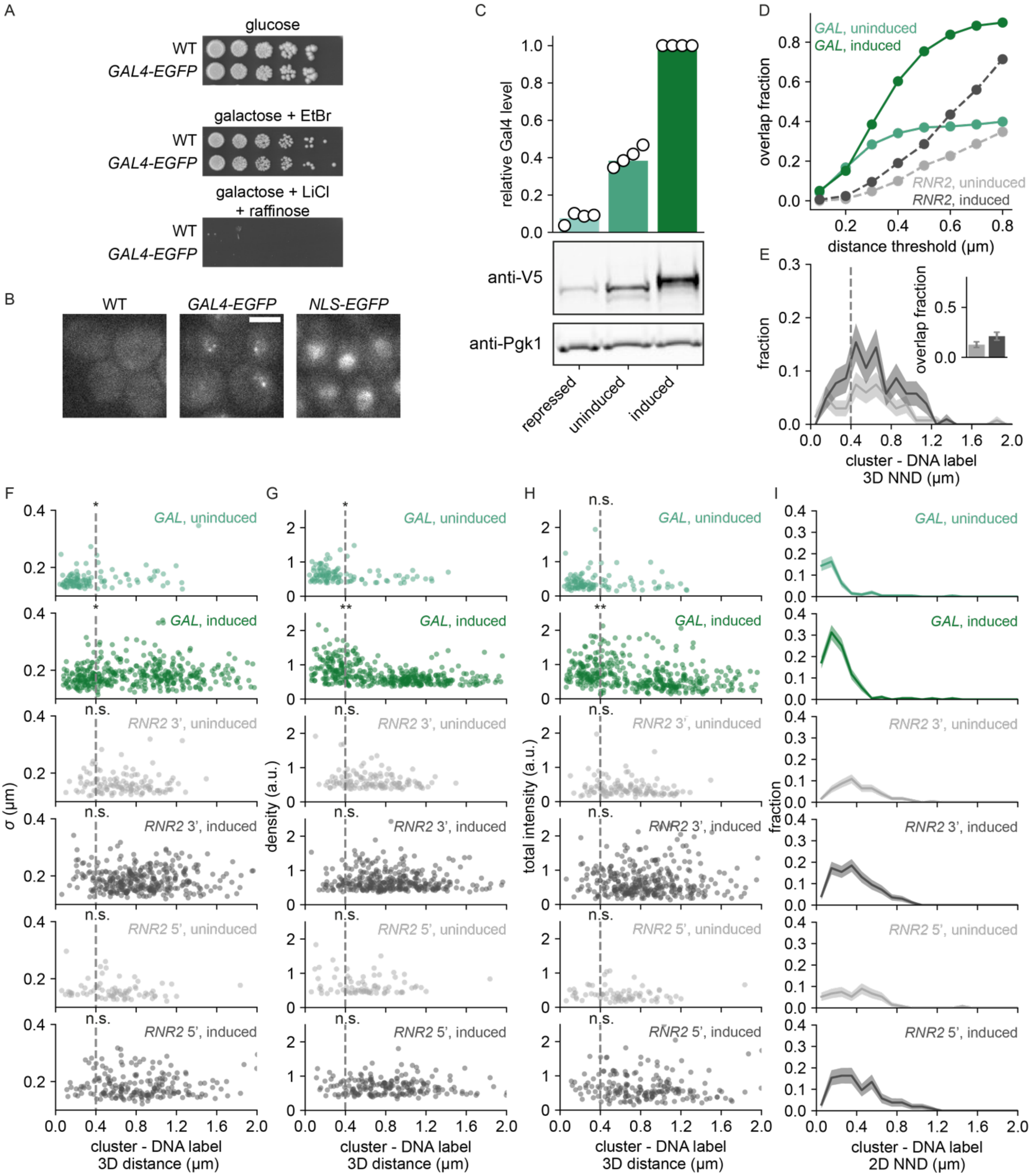

Figure S2. Gal4 forms clusters that colocalize with the *GAL* genes.

A. Growth assay of indicated strains to assess their galactose metabolism capability. Shown are 5-fold serial dilutions on YEP + 2% glucose (dilution control), YEP + 2% galactose + 20 μg/mL ethidium

bromide (growth = functional galactose metabolism) and YEP + 2% raffinose + 2% galactose + 40 mM lithium chloride + 0.003% methionine (no growth = functional galactose metabolism).

B. Representative images of WT yeast cells (left), cells expressing Gal4-EGFP (middle) and cells expressing NLS-EGFP in induced (galactose and raffinose) conditions. Only for Gal4-EGFP clustering is observed indicating the specificity of Gal4 for this clustering. Images are a single *z*-slice of a representative group of cells. Scalebar: 3 µm.

C. Western blot quantification of Gal4-EGFP-V5 protein levels using an anti-V5 antibody measured in repressed (glucose), uninduced (raffinose) or induced (galactose) conditions. Expression levels are normalized to Pgk1 and to the expression level in 2.00% galactose. Open circles represent the results of individual replicate experiments, green bars indicate their mean. Western blot images are a representative example of 4 independent experiments.

D. Fraction of cells with an overlapping cluster with the *GAL* DNA label in uninduced (raffinose, light green) and induced (galactose and raffinose, dark green) conditions or with the *RNR2* DNA label in uninduced (light grey) and induced (dark grey) conditions for varying distance thresholds used to discriminate overlapping and non-overlapping clusters. At all distance thresholds, the *GAL* DNA labels show more overlapping clusters than the negative control gene, in induced and uninduced conditions.

E. Distribution of 3D nearest neighbor distances (NNDs) between the *RNR2* 5' DNA label and the closest cluster in uninduced (light grey, 172 cells) and induced (dark grey, 118 cells) conditions. Shaded regions represent SEM based on 1000 bootstrap repeats. Vertical dashed line indicates 400 nm threshold used to discriminate between overlapping and non-overlapping clusters. Inset shows fraction of DNA-label containing cells with an overlapping cluster, with 13 $\pm$ 3% and 21 $\pm$ 4% overlap in uninduced and induced conditions, respectively. Error bars represent SEM based on 1000 bootstrap repeats.

F-H. Scatterplot of F. cluster σ, G. density and H. total intensity versus 3D distance between the Gal4-EGFP cluster and the *GAL* DNA label in uninduced (light green, 306 cells) and induced (dark green, 200 cells) conditions, the *RNR2* 3' DNA label in uninduced (light grey, 276 cells) and induced (dark grey, 198 cells) conditions, and the *RNR2* 5' DNA label in uninduced (light grey, 172 cells) and induced (dark grey, 118 cells) conditions. Vertical dashed line indicates 400 nm threshold used to discriminate between overlapping and non-overlapping clusters. Significance between cells closer and further than 400 nm from the DNA label was determined by Mann-Whitney *U* test; n.s.: not significant; *: $p < 0.05$; **: $p < 0.01$.

I. Distribution of 2D nearest neighbor distances (NND) between DNA label and the closest cluster for the same dataset and conditions as **F-H.** Shaded regions represent SEM based on 1000 bootstrap repeats.

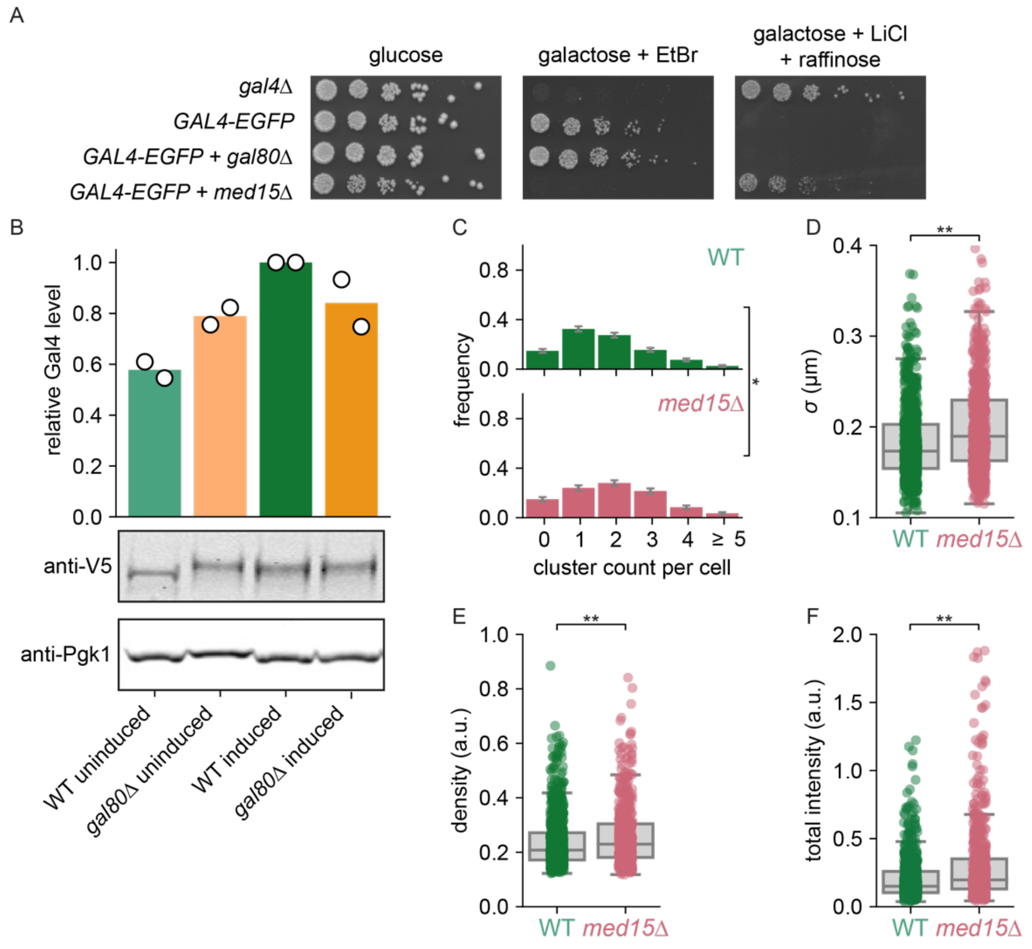

Figure S3. Gal4 clusters are limited by Gal80 but do not depend on interactions with Med15.

A. Growth assay of indicated strains to assess their galactose metabolism capability. Shown are 5-fold serial dilutions on YEP + 2% glucose (dilution control), YEP + 2% galactose + 20 µg/mL ethidium bromide (growth = functional galactose metabolism) and YEP + 2% raffinose + 2% galactose + 40 mM lithium chloride + 0.003% methionine (no growth = functional galactose metabolism).

B. Top: western blot quantification of Gal4-EGFP-V5 protein levels using an anti-V5 antibody measured in WT and *gal80Δ* in uninduced (raffinose) and induced (galactose) conditions. Expression levels are normalized to Pgk1 and to the expression level of WT in induced conditions. Open circles represent the results of individual replicate experiments, green and orange bars indicate their mean. Bottom: corresponding western blot image, representative example of two independent experiments.

C-F. Quantification of Gal4-EGFP clusters in WT (green, 484 cells) and *med15Δ* (pink, 372 cells) in induced (galactose and raffinose) conditions. **C.** Distribution of number of clusters observed per cell. Error bars indicate SEM based on 1000 bootstrap repeats; *: $p<0.05$. **D-F.** Distribution of **D.** cluster σ, **E.** density and **F.** total intensity. Circles show data for individual clusters and box plots show the distribution of the data, with box edges indicating first and third quartiles, center line indicating the median and whiskers indicating the 1.5x interquartile range. Significance determined by Mann-Whitney *U* test; *: $p<0.05$; **: $p < 0.01$.

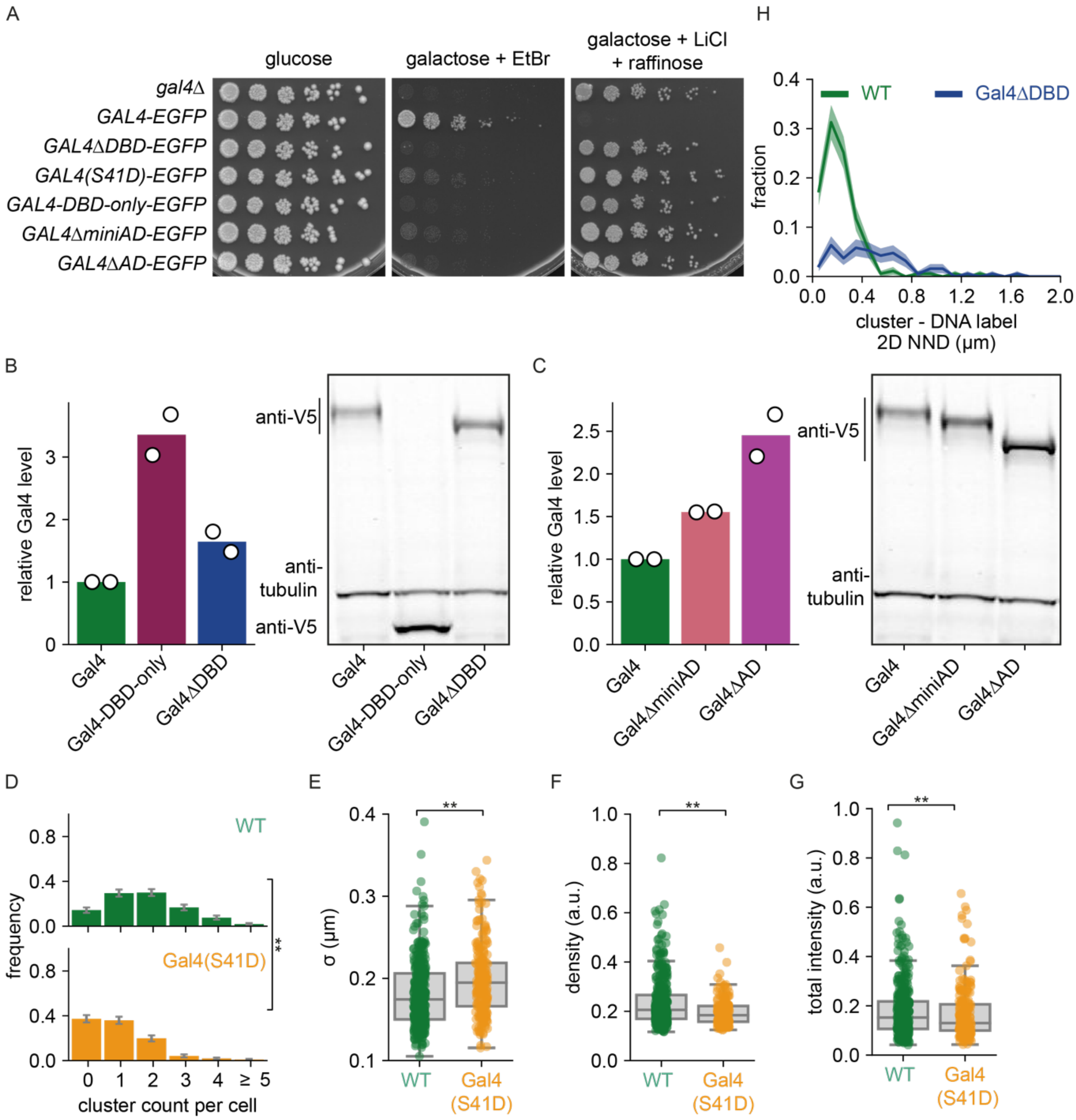

Figure S4. Characterization of different Gal4 mutants.

A. Growth assay of indicated strains to assess their galactose metabolism capability. Shown are 5-fold serial dilutions on YEP + 2% glucose (dilution control), YEP + 2% galactose + 20 μg/mL ethidium bromide (growth = functional galactose metabolism) and YEP + 2% raffinose + 2% galactose + 40 mM lithium chloride + 0.003% methionine (no growth = functional galactose metabolism).

B. Left: western blot quantification of Gal4-EGFP-V5, Gal4-DBD-only-EGFP-V5 and Gal4ΔDBD-EGFP-V5 protein levels using an anti-V5 antibody measured in induced (galactose and raffinose) conditions. Expression levels are normalized to tubulin and to the expression level of WT Gal4-EGFP-V5. Open circles represent the results of individual replicate experiments, colored bars indicate their mean. Right: corresponding western blot image, representative example of two independent experiments.

C. Left: western blot quantification of Gal4-EGFP-V5, Gal4ΔminiAD-EGFP-V5 and Gal4ΔAD-EGFP-V5 protein levels using an anti-V5 antibody measured in induced conditions. Expression levels are normalized to tubulin and to the expression level of WT Gal4-EGFP-V5. Open circles represent the results of individual replicate experiments, colored bars indicate their mean. Right: corresponding western blot image, representative example of two independent experiments.

D-G. Quantification of WT Gal4-EGFP (green, 210 cells) and Gal4(S41D)-EGFP (orange, 217 cells) clusters in induced (galactose and raffinose) conditions **D.** Distribution of number of clusters observed per cell. Error bars indicate SEM based on 1000 bootstrap repeats. **E-G.** Distribution of **E.** cluster σ, **F.** density and **G.** total intensity. Circles show data for individual clusters and box plots show the distribution of the data, with box edges indicating first and third quartiles, center line indicating the median and whiskers indicating the 1.5x interquartile range. Significance determined by Mann-Whitney *U* test; *: $p < 0.05$; **: $p < 0.01$.

H. Distribution of 2D nearest neighbor distances (2D NND) between the *GAL* DNA label and the closest WT Gal4-EGFP (green) or Gal4ΔDBD-EGFP (blue) cluster. Shaded regions represent SEM based on 1000 bootstrap repeats.

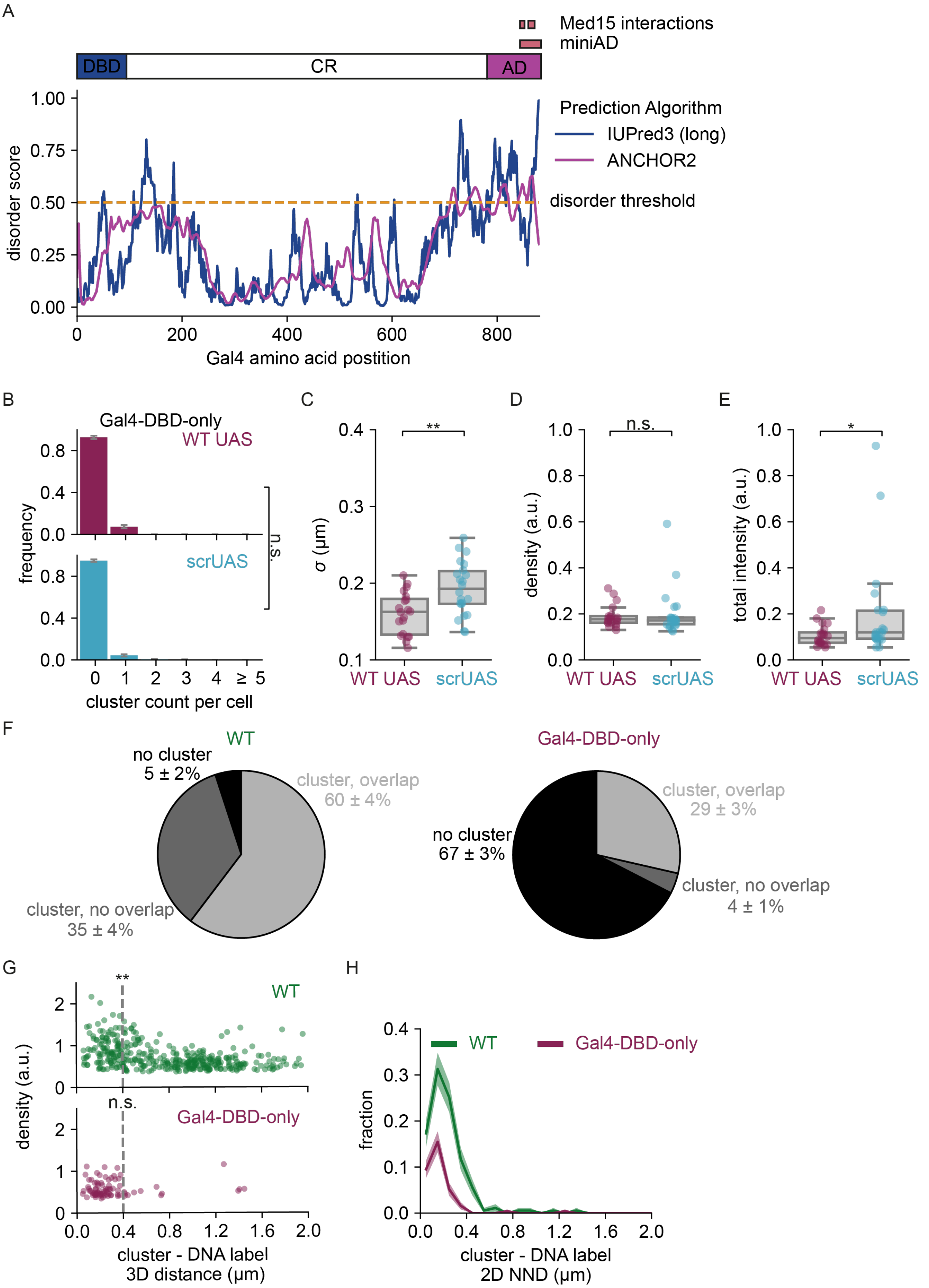

Figure S5. IDRs are not essential for clustering but contribute to target search.

A. Top: schematic representation of Gal4 protein domains as used in this study: DNA-binding domain (DBD; blue), Central Region (CR; white) and Activation Domain (AD; purple) with the miniAD and Med15 interaction domains (1) annotated (pink). Bottom: Predicted disorder of the Gal4 protein based on IUPred3 long (blue) and ANCHOR2 (purple) prediction algorithms (2, 3). Orange dashed line indicates disorder threshold of 0.5 to discriminate ordered from disordered regions.

B-E. Quantification of Gal4-DBD-only-EGFP clusters in cells with WT UAS (purple, 274 cells) and scrUAS (cyan, 348 cells) in induced (galactose and raffinose) conditions. **B.** Distribution of number of clusters observed per cell. Error bars indicate SEM based on 1000 bootstrap repeats. C-E. Distribution of **C.** cluster σ, **D.** density and **E.** total intensity. Circles show data for individual clusters and box plots show the distribution of the data, with box edges indicating first and third quartiles, center line indicating the median and whiskers indicating the 1.5x interquartile range. Significance determined by Mann-Whitney *U* test; n.s.: not significant; *: $p < 0.05$; **: $p < 0.01$.

F. Pie-charts showing the percentages of cells with a *GAL* DNA label that show a cluster overlapping with the *GAL* locus (light grey), a cluster that does not overlap with the *GAL* locus (dark grey), or do not show any clusters (black) for WT Gal4-EGFP (left, 179 cells) and Gal4-DBD-only-EGFP (right, 252 cells) in induced (galactose and raffinose) conditions. Error values are SEM based on 1000 bootstrap repeats.

G. Scatterplot of cluster density versus 3D distance between the cluster and the *GAL* DNA label for WT Gal4-EGFP (green, 200 cells) and Gal4-DBD-only-EGFP (purple 276, cells) in induced (galactose and raffinose) conditions. Vertical dashed line indicates 400 nm threshold used to discriminate between overlapping and non-overlapping clusters. Significance between clusters closer and further than 400 nm from the DNA label was determined by Mann-Whitney *U* test; n.s.: not significant; *: $p < 0.05$; **: $p < 0.01$.

H. Distribution of 2D nearest neighbor distances (2D NND) between the *GAL* DNA label and the closest cluster for WT Gal4 (green, 200 cells) and Gal4-DBD-only (purple, 276 cells), same dataset as in **G.** Shaded regions represent SEM based on 1000 bootstrap repeats.

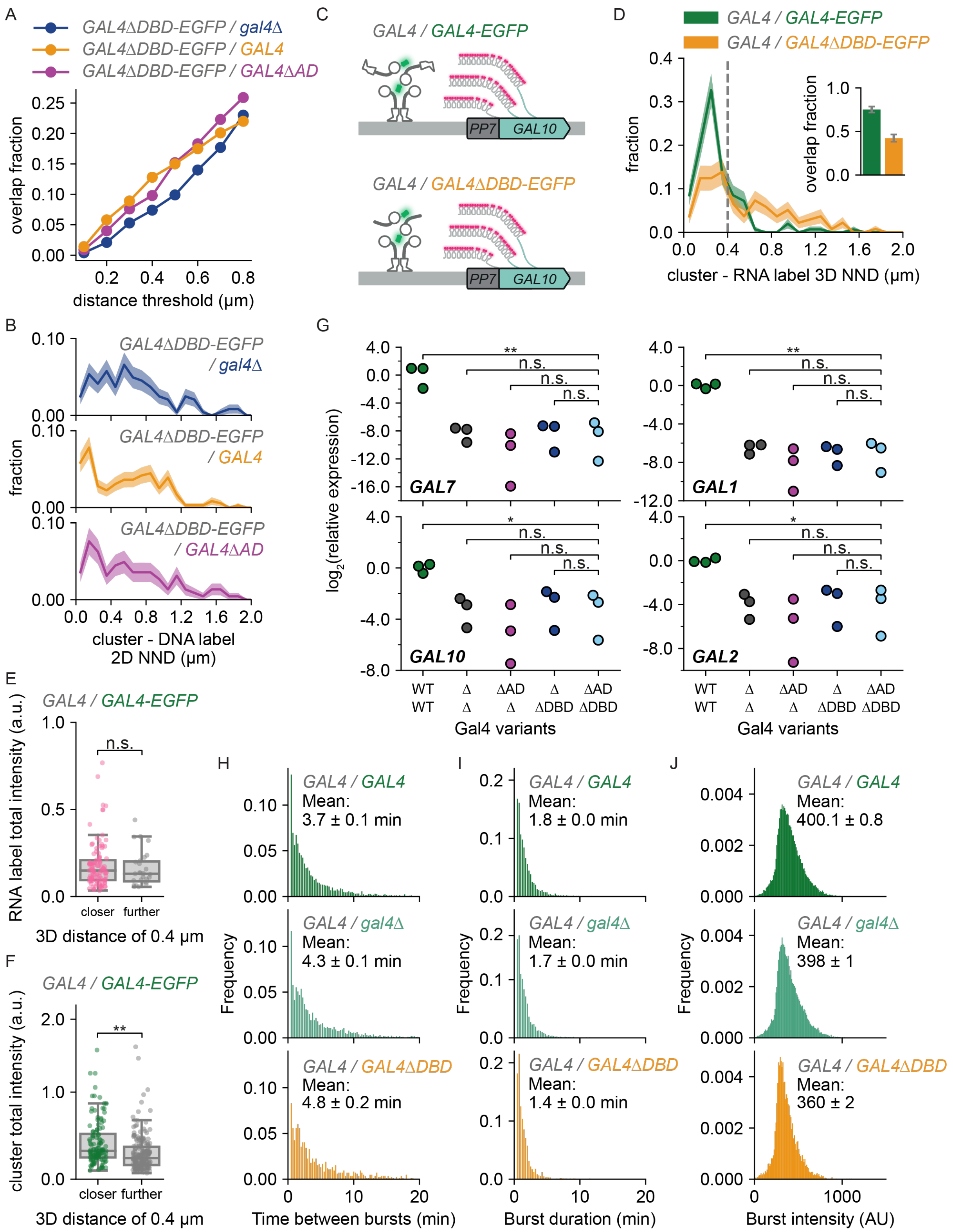

Figure S6. Gal4 self-interactions are sufficient to recruit Gal4 to target genes but insufficient to activate transcription.

A. Fraction of cells with a Gal4ΔDBD-EGFP cluster overlapping with the *GAL* DNA label for *GAL4ΔDBD-EGFP/gal4Δ* (blue, 300 cells), *GAL4ΔDBD-EGFP/GAL4* (orange, 423 cells) and *GAL4ΔDBD-EGFP/GAL4ΔAD* (purple, 256 cells) for varying distance thresholds used to discriminate

between overlapping and non-overlapping clusters. Addition of WT Gal4 or Gal4ΔAD results in increased overlap of Gal4ΔDBD clusters with the *GAL* DNA label regardless of the chosen threshold. Cells were grown in uninduced conditions (raffinose) and imaged after 30 minutes of induction (galactose and raffinose).

B. Distribution of 2D nearest neighbor distances (2D NND) between the *GAL* DNA label and the closest cluster for *GAL4ΔDBD-EGFP/gal4Δ* (blue, 300 cells), *GAL4ΔDBD-EGFP/GAL4* (orange, 423 cells) and *GAL4ΔDBD-EGFP/Gal4ΔAD* (purple, 256 cells) in induced conditions. Shaded regions represent SEM based on 1000 bootstrap repeats.

C. Schematic representation of the *PP7-GAL10* gene and diploid yeast strains expressing WT Gal4 from one allele and on the other allele expressing either WT Gal4-EGFP (*GAL4/GAL4-EGFP,* green) or Gal4ΔDBD-EGFP (*GAL4/GAL4ΔDBD-EGFP*, orange*)*. Nascent *GAL10* transcripts are fluorescently labeled by binding of the PP7-coat protein-ymScarletI to the PP7-stem loops (magenta), forming bright RNA label spots indicating the location of the *GAL10* transcription site and thereby the *GAL* locus.

D. Distribution of 3D nearest neighbor distances (NND) between the *PP7-GAL10* RNA label and the closest Gal4-EGFP (green, 161 cells) or Gal4ΔDBD-EGFP cluster (blue, 141 cells) in the presence of WT Gal4. Cells were grown in uninduced conditions (raffinose) and imaged after 30 minutes of induction (galactose and raffinose). Shaded regions represent SEM based on 1000 bootstrap repeats. Vertical dashed line indicates 400 nm threshold used to discriminate between overlapping and non-overlapping clusters. Inset shows fraction of RNA label-containing cells with an overlapping cluster. Error bars represent SEM based on 1000 bootstrap repeats.

E. Distributions of the total intensity of the *GAL10* RNA labels in *GAL4/GAL4-EGFP* yeast cells (same dataset as **D.**) with a cluster closer than 400 nm of the label (pink, 105 cells) or further (grey, 27 cells). Circles show data for individual RNA labels and box plots show the distribution of the data, with box edges indicating first and third quartiles, center line indicating the median and whiskers indicating the 1.5x interquartile range. Significance determined by Mann-Whitney *U* test; n.s.: not significant.

F. Distributions of total intensity of the Gal4-EGFP clusters in *GAL4/GAL4-EGFP* yeast cells (161 cells, same dataset as **D.**) closer than 400 nm from the *GAL10* RNA label (green) or further (grey). Circles show data for individual clusters and box plots show the distribution of the data, with box edges indicating first and third quartiles, center line indicating the median and whiskers indicating the 1.5x interquartile range. Significance determined by Mann-Whitney *U* test; **: $p < 0.01$.

G. Quantification of *GAL7*, *GAL1*, *GAL10* and *GAL2* mRNA levels measured by RT-qPCR in diploid yeast strains with different genetic backgrounds: *GAL4/GAL4* (WT/WT, green), *gal4Δ/gal4Δ* (Δ/Δ, grey), *GAL4ΔAD/gal4Δ* (ΔAD/Δ, pink), *gal4Δ/GAL4ΔDBD* (Δ/ΔDBD, dark blue) and *GAL4ΔAD/GAL4ΔDBD* (ΔAD/ΔDBD, light blue) with three biological replicates each. Cells were grown in induced (galactose and raffinose) conditions. Colored dots show replicate ΔC_q values compared to reference gene *ACT1*, normalized to the average of the ΔC_q values of the *GAL4/GAL4* (WT/WT)

strain. Significance between the ΔC_q values of the *GAL4ΔAD/GAL4ΔDBD* strain and the other strains was determined using Student's *t* test; n.s.: not significant; *: $p < 0.05$; **: $p < 0.01$.

H-J. Distributions of **H.** the time between bursts, **I.** burst duration and **J.** burst intensity for *PP7-GAL10* transcription in *GAL4*/*GAL4* (green, 673 cells), *GAL4/gal4Δ* (light green, 669 cells) and *GAL4/GAL4ΔDBD* (orange, 265 cells) yeast strains (same dataset as Figure 6D-H). Values indicated in the graph are the mean with standard deviation based on 1000 bootstrap repeats.

Supplementary Table 1: Yeast strains used in this study

Strain	Genotype	Source
BY4741	MATa *his3Δ1 leu2Δ0 met15Δ0 ura3Δ0*	Euroscarf
BY4742	MATα *his3Δ1 leu2Δ0 lys2Δ0 ura3Δ0*	Euroscarf
BY4743	MATa/α *his3Δ1/his3Δ1 leu2Δ0/leu2Δ0 LYS2/lys2Δ0 met15Δ0/MET15 ura3Δ0/ura3Δ0*	Euroscarf
YTL390	BY4742 with *GAL4-EGFP*	This study
YTL1702	BY4742 with *GAL4::BPSV40-EGFP*	This study
YTL762	BY4742 with *GAL4-EGFP* + *gal80Δ*	This study
YTL1304	BY4742 with *GAL4-EGFP* + *med15Δ*	This study
YTL1284	BY4742 with *BPSV40-GAL4(Δ1-94)-EGFP*	This study
YTL945	BY4742 with *GAL4(S41D)-EGFP*	This study
YTL1221	BY4742 with *GAL4(Δ840-881)-EGFP*	This study
YTL1286	BY4742 with *GAL4(Δ768-881)-EGFP*	This study
YTL1226	BY4742 with *GAL4(Δ95-881)-EGFP*	This study
YTL1653	BY4742 with *GAL4-EGFP-3xV5*	This study
YTL1685	BY4742 with *GAL4-EGFP-3xV5* + *gal80Δ*	This study
YTL1655	BY4742 with *BPSV40-GAL4(Δ1-94)-EGFP-3xV5*	This study
YTL1661	BY4742 with *GAL4(Δ840-881)-EGFP-3xV5*	This study
YTL1654	BY4742 with *GAL4(Δ95-881)-EGFP-3xV5*	This study
YTL559	BY4742 with *gal4Δ*	This study
YTL1154	BY4742 with *scrUAS 5'GAL2* + *scrUAS 5'GAL7* + 3x *scrUAS 5'GAL10* + *GAL4-EGFP*	This study
YTL1639	BY4742 with *scrUAS 5'GAL2* + *scrUAS 5'GAL7* + 3x *scrUAS 5'GAL10* + *GAL4(Δ95-881)-EGFP*	This study
YTL1652	BY4742 with *tetOx128 3'-GAL1 + ade1::tetR1-tdTomato-kanMX* + *GAL4-EGFP*	This study
YTL1698	BY4742 with *tetOx128 5'-RNR2 + ade1::tetR1-tdTomato-kanMX* + *GAL4-EGFP*	This study
YTL1699	BY4742 with *tetOx128 3'-RNR2 + ade1::tetR1-tdTomato-kanMX* + *GAL4-EGFP*	This study
YTL1662	BY4742 with *tetOx128 3'-GAL1 + ade1::tetR1-tdTomato-kanMX* + *BPSV40-GAL4(Δ1-94)-EGFP*	This study
YTL1686	BY4742 with *tetOx128 3'-GAL1 + ade1::tetR1-tdTomato-kanMX* + *GAL4(Δ95-881)-EGFP*	This study
YTL1679	BY4743 with *GAL1/tetOx128 3'-GAL1 + ADE1/ade1::tetR1-tdTomato-kanMX* + *BPSV40-GAL4(Δ1-94)-EGFP/gal4Δ*	This study
YTL1678	BY4743 with *GAL1/tetOx128 3'-GAL1 + ADE1/ade1::tetR1-tdTomato-kanMX* + *BPSV40-GAL4(Δ1-94)-EGFP/GAL4*	This study
YTL1693	BY4743 with *GAL1/tetOx128 3'-GAL1 + ADE1/ade1::tetR1-tdTomato-kanMX* + *BPSV40-GAL4(Δ1-94)-EGFP/GAL4(Δ768-881)*	This study
YTL1218	BY4743 with *GAL10/14xPP7-GAL10 + ura3Δ0/ura3Δ0::pRPL15A-PCP-ymScarletI* + *GAL4/GAL4-EGFP*	This study
YTL1317	BY4743 with *GAL10/14xPP7-GAL10 + ura3Δ0/ura3Δ0::pRPL15A-PCP-ymScarletI* + *GAL4/BPSV40-GAL4(Δ1-94)-EGFP*	This study
YTL1098	BY4743 with *GAL10/14xPP7-GAL10 + ura3Δ0/ura3Δ0::pRPL15A-PCP-ymScarletI* + *GAL4-EGFP/GAL4-EGFP*	This study
YTL1326	BY4743 with *GAL10/14xPP7-GAL10 + ura3Δ0/ura3Δ0::pRPL15A-PCP-ymScarletI* + *GAL4(Δ768-881)-EGFP/BPSV40-GAL4(Δ1-94)-EGFP*	This study

YTL590	BY4743 with *GAL10/14xPP7-GAL10 + ura3∆0/ura3∆0::pRPL15A-PCP-GFPEnvy*	This study
YTL1431	BY4743 with *GAL10/14xPP7-GAL10 + ura3∆0/ura3∆0::pRPL15A-PCP-GFPEnvy + GAL4/gal4Δ*	This study
YTL1432	BY4743 with *GAL10/14xPP7-GAL10 + ura3∆0/ura3∆0::pRPL15A-PCP-GFPEnvy + GAL4/BPSV40-GAL4(∆1-94)*	This study
YTL1834	BY4743 with *gal4∆/gal4∆*	This study
YTL1835	BY4743 with *GAL4(∆768-881)/gal4∆*	This study
YTL1836	BY4743 with *BPSV40-GAL4(Δ1-94)/gal4∆*	This study
YTL1837	BY4743 with *GAL4(∆768-881)/BPSV40-GAL4(Δ1-94)*	This study

Supplementary Table 2: Plasmids used in this study

Plasmid name	Source
pTL014: pURA pGAL CRE recombinase (Euroscarf pSH47)	(4)
pTL031: 14x PP7 with loxP-kanMX-loxP (Addgene 189939)	(5)
pTL071: 1xEGFP tagging vector with loxP-kanMX-loxP	This study
pTL131: pML104 Cas9 guide RNA construct (Addgene 67638)	(6)
pTL174: pURA SIV pRPL15A PCP-NLS-EGFPEnvy (Addgene 189941)	This study
pTL306: pURA SIV pRPL15A PCP-NLS-ymScarlet-I	This study
pTL314: pML104 with guideRNA to GAL4 (ggcgcacttcggtttttctt)	This study
pTL350: pML104 with guideRNA to GAL4 (gctactctcccaaaaccaaa)	This study
pTL354: pML104 with guideRNA to pGAL2 (ccgcacggacgaaagaccgc)	This study
pTL355: pML104 with guideRNA to pGAL7 (ttcggagcactgttgagcga)	This study
pTL363: pML104 with guideRNA to GAL4 (ggtgaaggccctactgagcc)	(7)
pTL370: pURA pADE3 BPSV40-EGFP	This study
pTL387: pML104 with guideRNA to GAL4 (atatacatcatccattgtag)	This study
pTL406: pML104 with gRNA to MED15 (taccctcggcacatttttct)	This study
pTL433: pML104 with gRNA to EGFP (ggatgaattgtacaaataac)	This study
pTL536: pDD207 tetR1-tdTomato	(8)
pTL539: pDD220 NAT-tet	(8)
pTL541: pDD226 tetOx128	(8)
pTL551: pML104 with guideRNA to pTEF (gagattttgactgcaatttc)	This study

Supplementary Table 3: Oligos used in this study (ordered from Integrated DNA Technologies)

Oligo name	Sequence
GAL4-del-F	tgggacttgaacagctcctt
GAL4-del-R	tttgggtgtcttcatcacca
GAL4-EGFP-F	gatgatgtatataactatctattcgatgatgaagataccccaccaaacccaaaaaaagaggccg ctctagaactagtggatcc
GAL4-EGFP-R	atggtgcacgatgcacagttgaagtgaacttgcggggtttttcagtatctacgattcattagctgggt accgcataggccac
GAL10-14PP7-5'-F	tattaaacttctttgcgtccatccaaaaaaaaagtaagaatttttgaaaattcaatataacaaagtgg gagcgaggagatcc

GAL10-5'-R	agcaccacctgtaaccaaaacaattttagaagtactttcactttgtaactgagctgtcatgcataggccactagtggatctg
GAL4UAS_construct1	aaaaccttctctttggaactttcagtaatacgcttaactgctcattgctatattgaagtaagccgacccagagggttaggagccgccgagccgctcacggaagactctcctccgtgcgtcctcgtcttcaccggtcgcgttcctgaaacgcagatgtgcaccgtgcctcccccgtgcgaacaataaagattctacaatactagcttttatggttatgaagaggaaaaattggcagtaa
GAL4(S41>D)	gctccaaagaaaaaccgaagtgcgccaagtgtctgaagaacaactgggagtgtcgctacgatcccaaaaccaaaaggtctccgctgactagggcacatctgacagaagtggaatcaaggc
pGAL2_UAS2_repair	gatctatattcgaaaggggcggttgcctcaggaaggcactggctccttggggcctctgcggagatatctgcgccgttcaggggtccatgtgccttggacg
pGAL7_UAS2_repair	ttcttaaattgctttgcctctccttttggaaagctatacttgcagtccatggagggtcaaggctcattagatatattttctgtcattttccttaacccaa
pGAL4_BPSV40-EGFP_F	gtgtctacgtaatgcacgccatcattttaagagaggacagagaagcaagcctcctgaaagatgaaaaggacagcagatgg
pGAL4_BPSV40-EGFP_R	atggtgcacgatgcacagttgaagtgaacttgcggggtttttcagtatctacgattcattttatttgtacaattcatccataccatgg
GAL4(del840-881)-linker-EGFP_insert	cggctagtaaaattgatgatggtaataattcaaaaccactgtcacctggtgccgctctagaactagtggatccgctgcaggaattcgatatcgtgtctaa
GAL4(del95-881)-linker-EGFP_insert	acatgattttgaaaatggattctttacaggatataaaagcattgttaacagccgctctagaactagtggatccgctgcaggaattcgatatcgtgtctaa
BPSV40-GAL4(del2-94)-EGFP_insert_R	aatctatctgtgacggcatctttattcacattatcttgtacaaataatccaacttttcttttctttttggagattc
Gal4(del768-881)-EGFP_insert	aacagctgcaatcattagtgccactgaccccgtctgctttgtttggtggcgccgctctagaactagtggatccgctgcaggaattcgatatcgtgtctaa
GAL10_tet_NAT_F	ttttgttattgtacgtggggcagttgacgtcttatcatatgtcaaagtcatttgcgaagtctgcattgacaagttgctacacg
GAL10_tet_NAT_R	aaaagtttcaagacggcaatctcttttactgcatctcgtcagttggcaacttgccaagactgagtgagtacagtacgtgacg
tetTargetL_pTL541_F	ctgcattgacaagttgctacacg
tetTargetR_pTL541_R	ctgagtgagtacagtacgtgacg
3'RNR2_tet_NAT_F	gagtatctctctatatatttctttttacgcagtctcttcaatctctttatctgcattgacaagttgctacacg
3'RNR2_tet_NAT_R	tatgattcagtgtatatatatatatataaataagaggtgcgaaagcccacctgagtgagtacagtacgtgacg
5'RNR2_tet_NAT_F	gcataggaagccgaagtcgaacaagaagcaggcaaagtttagagcactgcctgcattgacaagttgctacacg
5'RNR2_tet_NAT_R	gttgagaattttattttcctcctagttttttctttttttgagtgcggagggctgagtgagtacagtacgtgacg
insert_GAL4(del768-881)	aacagctgcaatcattagtgccactgaccccgtctgctttgtttggtggctaaaatgaatcgtagatactgaaaaaccccgcaagttcacttcaactgtg
GAL80::KANMX-F	tccttgccgaccagcgtatacaatctcgatagttggtttcccgttctttccactcccgtctatcgataccgtcgacctcg
GAL80::KANMX-R	ctcagtattcgtttttataacgttcgctgcactgggggccaagcacagggcaagatgcttctcgactagtggatctgata
med15del_insert	tgccgtactcaaagatcaaggattaaaacgctatttcttttaaatctgctacatttgaagtttccatactttgatacttttgaagttacttcgtttggt
Gal4-GFP-3V5_insert_F	gatggtccagtcttgttacc
Gal4-GFP-3V5_insert_R	gggagggcgtgaatgtaagcgtgacataactaattacatgactcgaccagttatggatctgtactatccagtcc
qPCR primers	Sequence
ACT1_2F	ctacgtttccatccaagccg
ACT1_2R	ccggccaaatcgattctcaa
GAL1_F	tgccgttgctttagctgttg
GAL1_R	tcttcctcaccgcaaacaga
GAL2_F	ggcgctttgacgttagttcc

GAL2_R	gccagccagtttttcagctt
GAL7_F	tctggccatttgagaccttg
GAL7_R	cgcattcaaaggagcctgat
GAL10B_F	acttgggttccggtaaaggt
GAL10B_R	gccatttcagttcgcgtttg

REFERENCES

1. Tuttle,L.M., Pacheco,D., Warfield,L., Wilburn,D.B., Hahn,S. and Klevit,R.E. (2021) Mediator subunit Med15 dictates the conserved ‘fuzzy’ binding mechanism of yeast transcription activators Gal4 and Gcn4. *Nat Commun*, **12**, 2220.

2. Erdős,G., Pajkos,M. and Dosztányi,Z. (2021) IUPred3: prediction of protein disorder enhanced with unambiguous experimental annotation and visualization of evolutionary conservation. *Nucleic Acids Res*, **49**, W297–W303.

3. Erdős,G. and Dosztányi,Z. (2020) Analyzing Protein Disorder with IUPred2A. *Current Protocols in Bioinformatics*, **70**, e99.

4. Lenstra,T.L., Coulon,A., Chow,C.C. and Larson,D.R. (2015) Single-Molecule Imaging Reveals a Switch between Spurious and Functional ncRNA Transcription. *Molecular Cell*, **60**, 597–610.

5. Donovan,B.T., Huynh,A., Ball,D.A., Patel,H.P., Poirier,M.G., Larson,D.R., Ferguson,M.L. and Lenstra,T.L. (2019) Live‐cell imaging reveals the interplay between transcription factors, nucleosomes, and bursting. *EMBO J*, **38**.

6. Laughery,M.F., Hunter,T., Brown,A., Hoopes,J., Ostbye,T., Shumaker,T. and Wyrick,J.J. (2015) New vectors for simple and streamlined CRISPR-Cas9 genome editing in Saccharomyces cerevisiae. *Yeast (Chichester, England)*, **32**, 711–720.

7. Brouwer,I., Kerklingh,E., Leeuwen,F. van and Lenstra,T.L. (2021) Dynamic epistasis analysis reveals how chromatin remodeling regulates transcriptional bursting. 10.1101/2021.12.15.472793.

8. Dovrat,D., Dahan,D., Sherman,S., Tsirkas,I., Elia,N. and Aharoni,A. (2018) A Live-Cell Imaging Approach for Measuring DNA Replication Rates. *Cell Rep*, **24**, 252–258.
